# Automated segmentation of soft X-ray tomography: Native cellular structure with submicron resolution at high-throughput for whole-cell quantitative imaging in yeast

**DOI:** 10.1091/mbc.E24-10-0486

**Published:** 2025-10-01

**Authors:** Jianhua Chen, Mary Mirvis, Axel Ekman, Bieke Vanslembrouck, Mark Le Gros, Carolyn Larabell, Wallace F. Marshall

**Affiliations:** ^a^National Center for X-ray Tomography, Lawrence Berkeley National Laboratory, Berkeley, CA 94720; ^b^X-ray Imaging Group, Experimental Facility Division, National Synchrotron Radiation Research Center, Hsinchu 300092, Taiwan; ^c^Department of Anatomy, University of California, San Francisco, San Francisco, CA 94158; ^d^Department of Biochemistry and Biophysics, University of California, San Francisco, San Francisco, CA 94158; Duke University

## Abstract

Soft X-ray tomography (SXT) is an invaluable tool for quantitatively analyzing cellular structures at suboptical isotropic resolution. However, it has traditionally depended on manual segmentation, limiting its scalability for large datasets. Here, we leverage a deep learning-based autosegmentation pipeline to segment and label cellular structures in hundreds of cells across three *Saccharomyces cerevisiae* strains. This task-based pipeline uses manual iterative refinement to improve segmentation accuracy for key structures, including the cell body, nucleus, vacuole, and lipid droplets, enabling high-throughput and precise phenotypic analysis. Using this approach, we quantitatively compared the three-dimensional (3D) whole-cell morphometric characteristics of wild-type, VPH1-GFP, and *vac14* strains, uncovering detailed strain-specific cell and organelle size and shape variations. We show the utility of SXT data for precise 3D curvature analysis of entire organelles and cells and detection of fine morphological features using surface meshes. Our approach facilitates comparative analyses with high spatial precision and statistical throughput, uncovering subtle morphological features at the single-cell and population level. This workflow significantly enhances our ability to characterize cell anatomy and supports scalable studies on the mesoscale, with applications in investigating cellular architecture, organelle biology, and genetic research across diverse biological contexts.

## Abstract (executive summary)

- SXT offers many powerful features for whole-cell multiorganelle imaging, but, like other high-resolution volumetric imaging modalities, it is typically limited by low-throughput due to laborious segmentation.
- Autosegmentation on single-cell volumes for SXT overcomes this limitation, enabling statistical 3D morphometric analysis of multiple organelles in whole cells across cell populations.
- The combination of high 3D resolution of SXT data with statistically useful throughput represents an avenue for more thorough characterizations of cells in toto and opens new mesoscale biological questions and statistical whole-cell modeling of organelle and cell morphology, interactions, and responses to perturbations.

## INTRODUCTION

The cellular mesoscale, spanning large molecular complexes to the largest subcellular structures, including organelles and cell-wide cytoskeletal assemblies, is emerging as a new frontier in cell biology (Sear *et al.*, 2015; Ekman *et al.*, 2017; Goodsell *et al.*, 2020). At this scale of biological organization, bulk molecular activities culminate with bulk biophysical effects to produce highly complex cell-wide functions such as cell division, organelle trafficking, and metabolism coordinated across multiple compartments (Qian and Beltran, 2022). The immense complexity of the mesoscale requires novel tools, technologies, and analytical approaches to yield insights into how structure and function are coordinated at the whole-cell level.

The detailed study of intricate cellular structures through microscopy plays a central role in understanding the complex mechanisms governing cellular function and pathology. The budding yeast *Saccharomyces cerevisiae*, is an indispensable model organism in understanding molecular pathways involved in organelle size and shape determination, owing to its powerful genetic tractability and similarity to multicellular animals, including humans, combined with its amenability to imaging methods such as thin-section electron microscopy. *S. cerevisiae* has long been used as a primary model for studying myriad fundamental cellular processes, including, in recent years, the advent of the field of organelle–organelle interactions (Kornmann *et al.*, 2009; Hughes and Gottschling, 2012; Murley *et al.*, 2013; Elbaz-Alon *et al.*, 2014; Hariri *et al.*, 2018; Shai *et al.*, 2018, and many others). However, the small size of yeast cells and their organelles has made it a challenging system for light microscopy-based analysis of organelles. As a result, much of what is known about yeast cell architecture has come from thin-section transmission electron microscopy, a method that gives high resolution but at the cost of extremely tedious sample preparation, which effectively limits studies to either random sections or serial sections of a very small number of cells. Confocal fluorescence microscopy has been extensively used to image yeast cells and organelles, and by serving as the foundation for integration with other super-resolution imaging modalities, excellent spatial, and temporal resolution have been achieved. However, with all such confocal methods, the spatial resolution is anisotropic due to the asymmetric nature of the point spread function (PSF).

In contrast with the low-throughput of thin-section EM and the anisotropic spatial resolution of confocal microscopy, soft X-ray tomography (SXT) emerges as a powerful option, offering unparalleled advantages for the noninvasive and comparatively high-throughput imaging of cells. Unlike other methods, SXT does not require the introduction of external markers, therefore preserving the native state of the cell. This imaging modality, which uses low-energy X-rays, generates high-contrast 3D images of the cells based on the differential X-ray absorption properties of subcellular components. Because the absorption is linear, quantitative linear absorption coefficient (LAC) values are generated that reflect compositional and density variations of structures at the single-cell level. By using full-rotation tomography, this LAC is faithfully reconstructed to near-isotropic resolution, such that the image reflects the compositional density and structures at a single-cell level in a quantitative way. These unique capabilities are exceptionally valuable for quantitatively assessing the morphological and structural changes in yeast cells, thereby differentiating their phenotypic alterations. Importantly, multiple yeast cells can be imaged in their entirety in less than 10 min (Parkinson *et al.*, 2008; Le Gros *et al.*, 2014; Do *et al.*, 2015). The ability to image entire cells is important because it allows the full organelle complement of each cell to be visualized and quantified, along with information about the overall cell volume and surface that would be impossible to determine from small subimages of a cell, typical of other high-resolution methods like cryo-EM.

The ability to rapidly collect whole-cell images from large numbers of cells in a single experiment leads to a new challenge—how to identify the individual organelles from each label-free 3D image dataset. This process of data segmentation, which enables researchers to isolate specific organelles and examine the structural parameters within the cell, is of critical importance for accurate segmentation in yeast cell biology, as it underpins all further analysis and interpretation of cellular morphology. It is particularly fundamental to any efforts to use SXT as a routine method for quantifying cell anatomy, defined as the cumulative morphology and positioning of several organelles in the whole cell context. The ability to accurately segment and analyze images of cells and their components observed from various modalities facilitates a deeper understanding of cellular functions, behaviors, and interactions (Loconte *et al.*, 2022; 2023). For high-resolution contrast-based microscopy modalities, especially volumetric modalities such as SXT, segmentation is typically done manually. This is an extremely laborious and time-intensive task, requiring hours, or in some cases, days, to segment a single cell, depending on the number of structures being segmented per image. Automating this bottleneck step of manual image segmentation allows for the rapid processing of large volumes of data. Autosegmentation leverages computational algorithms to dissect images into their fundamental components, delineating these segments based on their distinctive features and properties. This process facilitates the precise identification and quantification of subcellular structures in a high-throughput manner. This is particularly indispensable for current large-scale data-intensive approaches in cell science, in which analyses of large datasets are increasingly required to draw meaningful conclusions.

The advancement of deep learning-based methods has shown great potential in achieving efficient and accurate autosegmentation (Pelt and Sethian, 2018; Ekman *et al.*, 2020; Nahas *et al.*, 2022; Egebjerg *et al.*, 2023; Dyhr *et al.*, 2023; Erozan *et al.*, 2024). Convolutional neural networks (CNN), especially U-net in its various forms, have proven highly effective in tasks such as medical image segmentation and object recognition (Fu *et al.*, 2021; Siddique *et al.*, 2021; Azad *et al.*, 2024), where their hierarchical and learned representations enable accurate and robust delineation of regions of interest. CNN offers a versatile, functional space that encompasses both the extracted features and the combinations with a single trainable optimization function.

In this work, we combine soft X-ray imaging, deep learning autosegmentation, genetic manipulation of yeast cells, and a robust method for analyzing and quantifying subcellular structures in whole cells, resulting in a workflow for large-scale analysis of cell anatomy at the whole-cell level. We describe the full process and technical considerations of developing a custom autosegmentation algorithm and demonstrate the potential for statistical analysis of organelle and cell morphometrics and biophysical properties across genetic conditions and detailed global and local shape analysis using meshing of isotropic volumes. The integration of quantitative analysis with high-resolution imaging aims to bridge the gap between cell biology and genetics, providing a holistic view of cellular anatomy and its functional implications, potentially paving the way for advancements in genetic engineering, therapeutic development, and beyond.

## RESULTS

### Comparing whole-cell reconstructions of budding yeast from confocal fluorescence imaging and SXT

**FIGURE 1:**
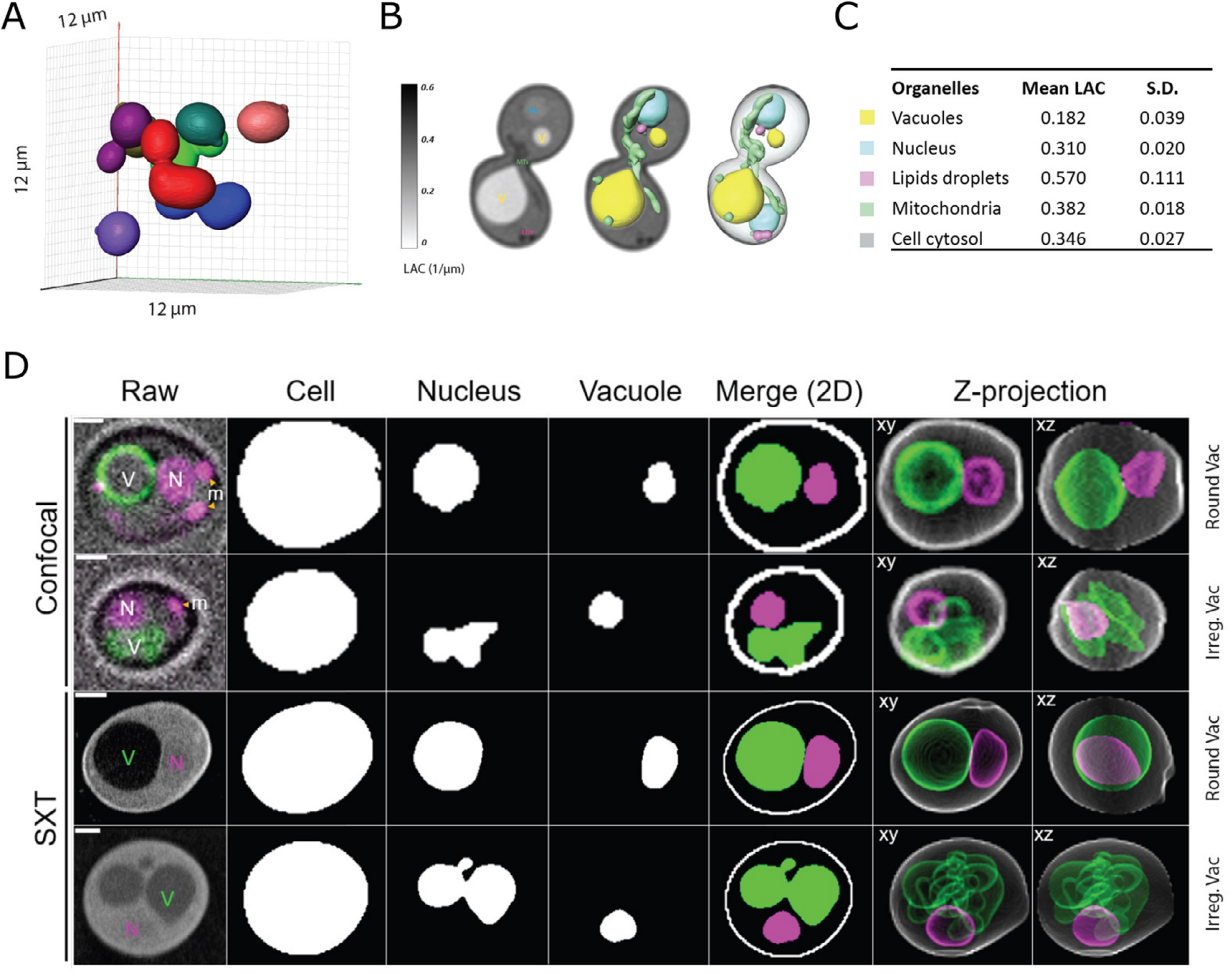
SXT imaging and segmentation of yeast cells. (A) 3D rendering of yeast cells in a single tomographic reconstruction. (B) Manual segmentation of organelles in single-cell tomograms based on LAC. (C) Mean and SDs of LACs of segmented organelles. (D) Examples of single yeast cell 3D raw data and segmentations. (i and ii) Confocal micrographs with vacuole (V) marked by VPH1-GFP and nucleus (N) marked by DAPI, which also stains mitochondrial DNA (m). (iii and iv) SXT raw tomograms. Segmented cell, nucleus, and vacuole are shown as representative 2D slices and sum slices z-projections of edge detections. 3D rotations of each cell are provided in Supplemental Movies S1–S4.

We generated 3D soft X-ray tomographic reconstructions of cryopreserved log-phase yeast cells based on organelle segmentations. Figure 1A shows the 3D surface rendering of multiple budding yeast cells at various stages of the cell cycle within a 12 × 12 × 12 µm field of view. This high-throughput imaging modality, relative to modalities of comparable resolution such as serial sectioning EM, with a data acquisition time of 5 min for eight yeast cells, nondestructively captures both the external contours of the entire cell and the internal organelle structures without the need for additional labeling, thereby revealing the morphologies in their native state. To quantify and analyze the biophysical properties of the cell structures, we then manually segmented the subcellular organelles based on their distinct morphologies and LACs. Figure 1B demonstrates the manual segmentation process of organelles in a dividing yeast cell, underscoring the ability of SXT to provide high-resolution, quantitative data that can be used to analyze morphology and composition accurately. Figure 1C depicts the LAC distribution across different organelles. The mean LAC, along with their SD, are provided for vacuoles, nuclei, lipid droplets (LD), mitochondria, and cytosol, which are 0.182 µm^–1^ (±0.039 µm^–1^), 0.310 µm^–1^ (±0.020 µm^–1^), 0.570 µm^–1^ (±0.111 µm^–1^), 0.382 µm^–1^ (±0.018 µm^–1^), and 0.346 µm^–1^ (±0.027 µm^–1^), respectively. These measurements allow for the differentiation of organelle compositional density and 3D spatial distribution.

As a proof of concept of the quality of image data and cellular information achievable in whole-cell SXT imaging, we show a side-by-side comparison of single-cell examples from standard confocal microscopy and SXT, including segmentations and 3D reconstructions of the cell, vacuole, and nuclear volumes for each cell (Figure 1D; Supplemental Movies S1–S4). Two representative cells from each modality, one with a spherical vacuole and one with a vacuole of irregular geometry, are displayed. The confocal data have a near-isotropic voxel size of 93 nm laterally (xy) and 100 nm axially (z), and the SXT data have an isotropic voxel size of 30 to 36 nm, with exact resolution varying slightly between individual tomograms across our dataset. SXT data were initially manually segmented based on the LAC, reflected in the visible contrast among structures in the raw tomogram. SXT has clear advantages for generating more realistic 3D representations of cells compared with confocal, providing more precise object boundaries for morphometric quantification, and revealing detailed shape information in complex structures such as clustered or irregularly shaped vacuoles. Although confocal deconvolution improves the distortion of optical data in the z-direction, SXT is nearly isotropic and does not show any distortion along any axis. The label-free nature of SXT of cells circumvents concerns of a nonspecific signal, the perturbative potential of fluorescent labels and phototoxicity, as well as possible shape distortions arising from organelle movements during live cell image acquisition. Thus, SXT offers superior 3D structural resolution, with multiorganelle information at the whole-cell level. However, the accessible structural information at this resolution lags behind optical modalities due to the limited throughput of manual segmentation.

### The application of machine learning and CNNs to segmentation

To date, most studies that quantitatively analyze cellular structures at suboptical resolution still heavily rely on manual labeling—a time-consuming, labor-intensive process that often depends on the individual's expertise in identifying features within the reconstruction. As we move toward high-throughput analysis, manual labeling becomes increasingly impractical due to the growing number of samples. With the emergence of promising automatic segmentation methods (Çiçek *et al.*, 2016; Pelt and Sethian, 2018), it is crucial to develop workflows that effectively integrate these tools into the structural analysis pipeline for large datasets. To meet this need, we have used a task-based segmentation pipeline. Each research question requires a specific set of semantic needs. We aim to create a pipeline that can collect the necessary data, choose an appropriate model architecture, and train a task-specific algorithm tailored to the user's specific inquiries. Here, we present an example of a fully 3D U-Net-type CNN.

**FIGURE 2:**
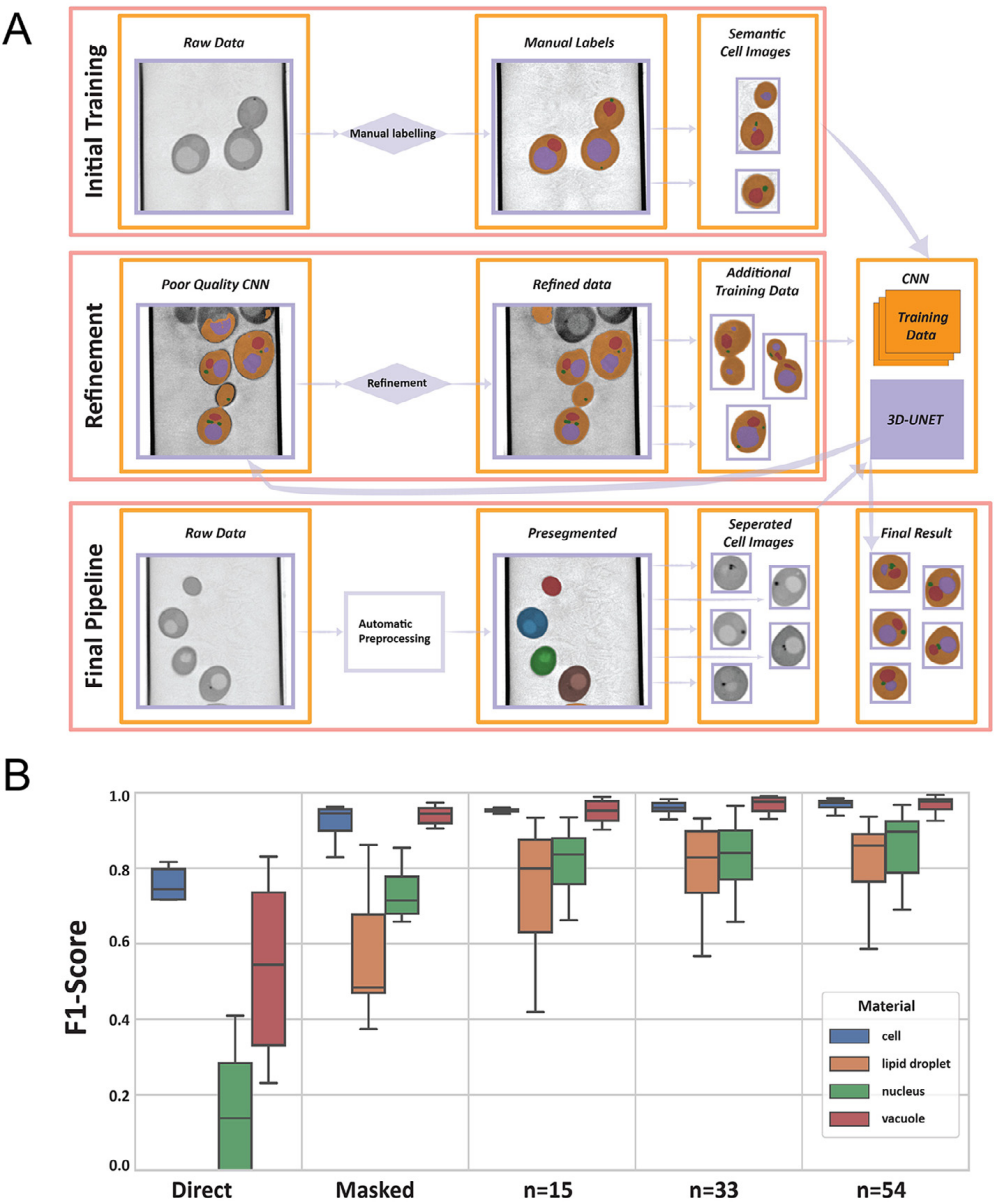
Overview of the iterative process of segmenting a batch of cell images. (A) An initial set of manual labels is used to train the model. Poor quality inferences from the initial model were manually corrected and added to the training data. This iterative refinement continues until the model achieves satisfactory segmentation quality. (B) Improvement of F1-Scores with manual iterative refinement showing the F1-scores for the different labels (cell, lipids, nucleus, and vacuole). The “Direct” column shows the initial F1-scores trained using full volume. The “Masked” column and subsequent columns show results of using presegmentation of the individual cells before training.

In the case of this work, the data were raw 3D SXT images of yeast cells, and the task was to use ML to aid in the segmentation of the individual cells and their semantic labeling into cell, nucleus, vacuole, and LDs. The outline of the iterative process is shown in Figure 2. We start with an initial set of manually labeled data (*n* = 7), from which an initial model is trained. This model is then used to generate automatic labels for the whole set of raw data from hundreds of cells. From this set of initial results, the user then selects poor-quality segmentations and either manually segments or refines them to be used as additional training data for the next iteration. A key element of our workflow is that the cells were first presegmented, allowing each individual cell to be segmented separately. This instance segmentation as a preprocessing step significantly accelerates the training process, reducing the size of each 3D input volume and ensuring that training data does not contain “empty” datasets, and the problem with partial cells (cells clipped by the boundary of the image) that can be difficult to label manually is avoided. The presegmentation involved identifying the cells within the capillary and using watershed segmentation for separation, and allowed for the use of partially labeled data (raw tomograms, where only one cell was manually labeled). The progression of this partially manual segmentation process with increasing numbers of cells is shown in Figure 2B. A key challenge lies in identifying the optimal stopping point. With too few samples, the generalization of the model to the dataset is poor. Conversely, excessively continuing the iterative process diminishes the method's advantage, essentially converging toward manually segmenting the entire dataset.

The metrics used to train the segmentation models do not directly address the usability of results in terms of the research question. Furthermore, the required loss metric of segmentation to answer research questions depends on the metrics that one wants to extract. Erozan *et al.* (2024) showed that a direct U-Net is well-suited for segmenting and assessing cell volume but can produce statistically significant differences in metrics such as surface area when compared with the training data, indicating the need for additional refinement or alternative methods. Volumetric measurements, such as total volume, mean LAC, or structural thickness, may not require as precise segmentations at the boundary, whereas surface area measurements and minimum distance-based metrics (like structural thickness) are highly sensitive to single voxel errors, which are not well captured by voxel-based loss functions.

**FIGURE 3:**
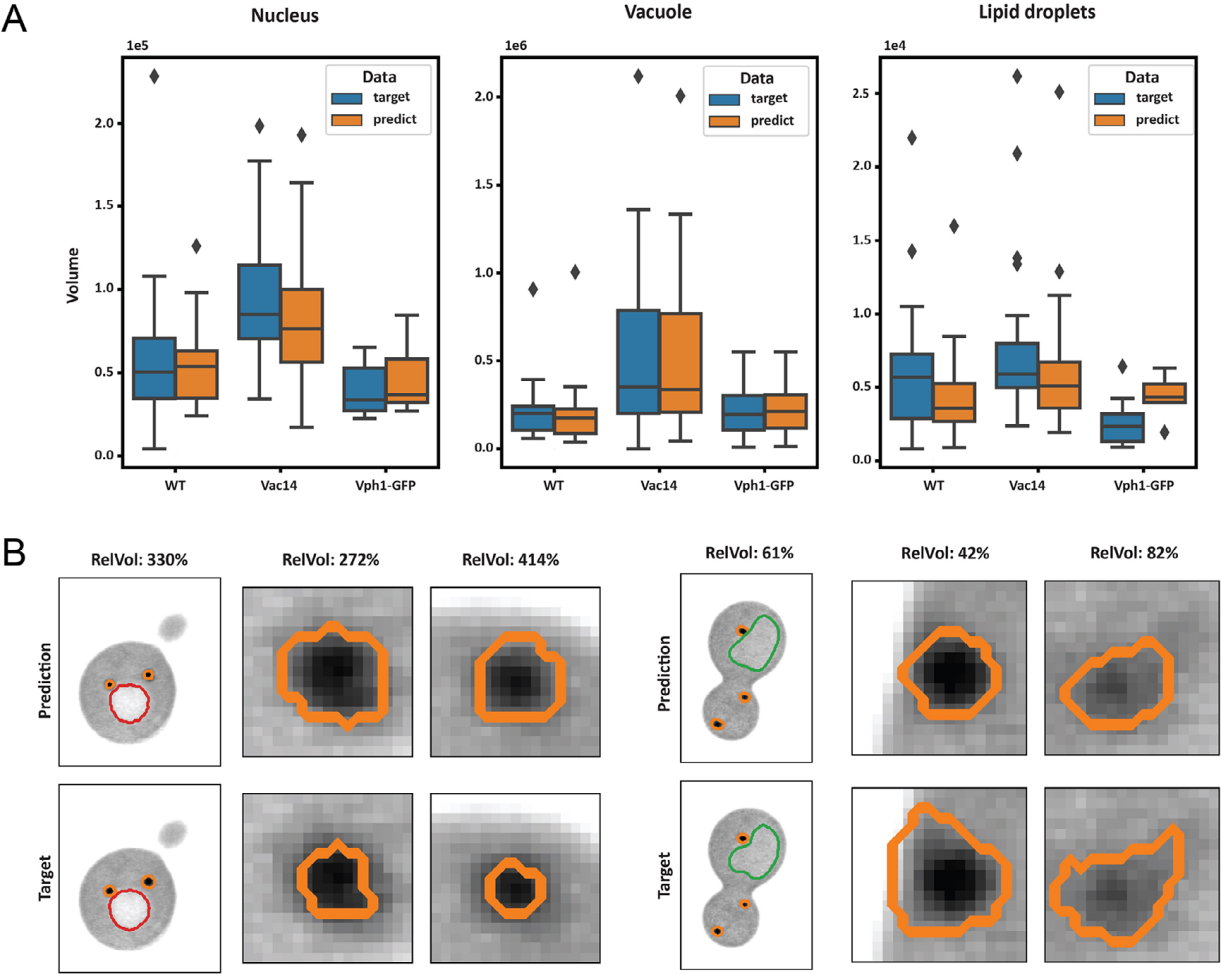
Validation of autosegmentation. (A) Volume measured from the training data and predictions, respectively, for the three different identified organelles. The volume predictions for the nucleus and vacuole do not deviate significantly from the manual labels, indicating that the model can reliably be used to describe population statistics of these cell structures. However, the segmentation of lipids shows considerable deviation from the manual data, suggesting that further refinement and quality assessment are needed for accurate lipid volume predictions. (B) Example slices of two outliers in the lipid volumes from population VPH1-GFP (left) and WT (right). Zoomed in slices show two examples of the labeled LDs (orange contour). RelVol depicts the relative volume of the predicted lipid label to the (manually segmented) target label. Red and green contours are of nucleus and vacuole, respectively.

In Figure 3A, we show the distribution of the label field volumes in both the training data and model predictions. Although the F1 score for the nucleus label is substantially lower than that of the vacuole, the automatic segmentation does not produce significantly different results in terms of volume when assessing population statistics. Conversely, for LD volumes in the case of VPH1-GFP, the automatic segmentation results show significant deviations from the manual labels, indicating that these results require further evaluation for reliability. Figure 3B provides examples where CNN has overestimated or underestimated the volume of LDs compared with the training data, highlighting the need for careful evaluation and potential refinement of the automatic segmentation results.

To evaluate the impact of iterative training on segmentation error and flexibility in terms of measurable features, we retrained a segmentation model as described, trained on 111 manually segmented cells. The models were trained in an iterative pipeline, initially with wild-type (WT) and *vac14* cells, and the final version trained on all 111 cells. In this final round of training, we introduced three new datasets *(fab1-2* temperature-sensitive mutant at permissive and restrictive temperatures *(fab1*^PT^ &*fab1*^RT^), and WT cells arrested with alpha-factor (aF), to test how the trained model would perform on additional samples. We chose samples that were biologically distinct but fundamentally structurally similar to the original training set because large deviation in morphology and LAC will limit the generalizability of the pipeline. Fab1p is a PI kinase upstream of Vac14p in the vacuole PI(3,5)P2 biosynthetic pathway, and *fab1-2* mutations cause extreme vacuole enlargement, only tolerated in temperature-sensitive mutants (Yamamoto *et al.,* 1995; Bonangelino *et al.,* 1997; Gary *et al.,* 1998). Therefore, *fab1-2*^PT^ is phenotypically similar to WT, while *fab1-2*^RT^ within, or at the extremes of, *vac14* phenotypic variation. aF-arrested cells should be generally phenotypically similar to WT, but with slightly altered polarized (“schmooing”) cell shape (Mackay and Manney, 1974).

We performed *k*-fold cross-validation (*k* = 3) for training. For each fold, models were trained using the samples from two subsets and validated on the remaining subset, which was used to monitor performance and apply early stopping. The final segmentation ensemble consists of the k independently trained models, and predictions were obtained by averaging their softmax outputs. All reported validation metrics were computed by aggregating predictions on the respective held-out folds to provide an evaluation across the entire training data without bias. After a cell was introduced to the training set, it was removed from the validation set. Cell, nucleus, vacuole, and LDs were segmented for all 111 cells for each training iteration, resulting in five segmentations per object: the manual segmentation (“target”), three intermediate models with 15, 53, and 78 cells (“*pipe_15″*, “*pipe_53″*, and “*pipe_78″*), and the final segmentation (“*pipe_111″*). We measured voxel-and mesh-based morphometrics on all five cell, vacuole, and nucleus segmentations for each cell and analyzed measurement evolution and overall error. As previously discussed, overall segmentation error is high for LDs, which were thus excluded from this analysis due to uncertainty in corresponding object identity between iterations.

Bulk error is shown for each voxel metric as a distribution of ratios of each measurement to the manually segmented target for all 111 cells (Supplemental Figure S1; Supplemental Table S1). Overall, final measurement error is low, generally within 5% for cell segmentation, 10 to 15% for nucleus, 5 to 10% in vacuole in all three metrics. In all cases, error decreases with successive rounds of training in final segmentations. Despite low error overall, cell volume error tends to be over- and underestimated equally, while cell surface area skews toward underestimation, and sphericity skews toward overestimation. Vacuole measurements show a wide variation in error in the initial model (*pipe_15*) but correct substantially with additional training data, with final segmentations similar in error to that of cells. We see no distinguishable difference between error for WT and *vac14* samples, which were the main training data for the model, and *fab1* and aF samples, which were added only in the final iteration (Supplemental Figure S1, prime (′) panels).

Examining nine individual representative cells, we find that the overall accuracy and degree of iterative improvement is variable between individual cells (Supplemental Figure S2; Supplemental Table S2). However, there is strong agreement between measurements from manual and autosegmentation for cell and vacuole metrics. We see lower accuracy in early training iterations for the largest vacuoles (*vac14*-1, *fab1* RT), but progressive improvement with subsequent iterations. Nucleus metrics showed significantly more variability, potentially due to the reduced contrast between nucleus and cytoplasmic LAC relative to cell boundaries and vacuoles, increasing the potential for segmentation error in both manual and automated segmentations. although in most cases the initial training (*pipe_15*) may result in a large over- or underestimate relative to target. Nevertheless, iterative segmentation generally improved the agreement between measurements from target and final segmentations. Overall, there was no obvious trend in the accuracy of measurements based on the iteration of training in which the cells were introduced - similar degrees of error and improvement is seen in cells introduced early and late in the pipeline, suggesting that introduction of new subsets of cells or even new cell types or cellular conditions all contribute to the iterative improvement of the model. After *pipe_53*, segmentation was reasonably accurate for fab1 and aF cells even though they were not included in the training. The largest error is seen in the *fab1* RT nucleus volume and surface area measurements, for which it was found that all segmentations result in measurements significantly smaller than the manually segmented target, although progressive iterations of training marginally improve the result. These results show robust automated segmentation across all strains, enabling quantitative comparative analysis of cell lines, only requiring a fraction of the collected data to be manually segmented.

### Quantitative phenotypic analysis from SXT data comparing autosegmented yeast strain morphometrics

**FIGURE 4:**
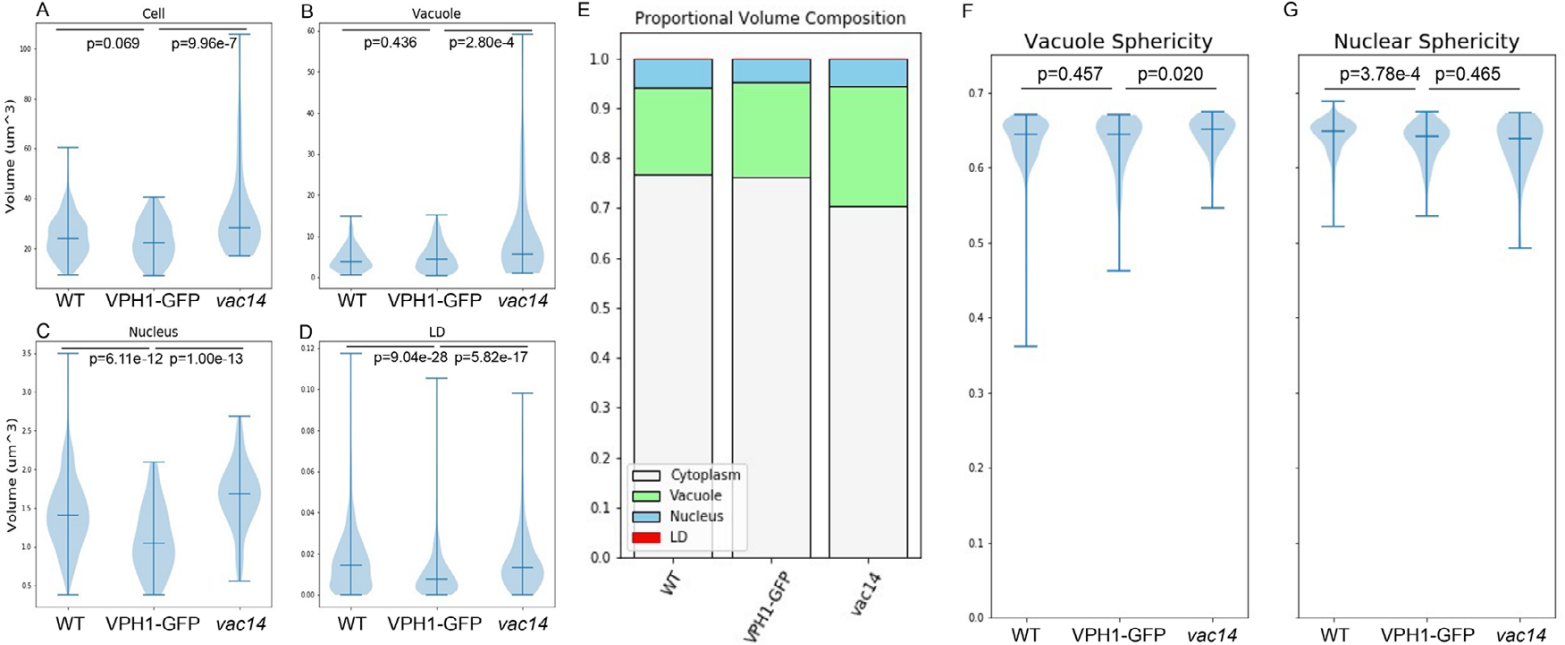
Quantitative phenotypic analysis from SXT data comparing autosegmented yeast strain morphometrics. Volume distributions for cell (A), vacuole (B), nucleus (C), and lipid (D) droplets plotted as violin plots for WT, VPH1-GFP, and *vac14* strains. Midline marks the median. (E) Proportional volume composition for each strain. (F) Vacuole and (G) nucleus sphericity distributions plotted as violin plots for each strain, with midline marking the median. *P* values reported from two-way Mann–Whitney *U* tests for nonparametric distributions. *N* = 287 (WT), 138 (VPH1-GFP), and 65 (*vac14*). Complete summary and significance test statistics reported in Supplemental Table S3.

We used the autosegmentation outputs in the form of voxel-based binary image stacks to carry out a statistical 3D morphometric characterization of cells and organelles in unbudded early G_1_ cells in three related strains: the haploid WT strain BY4741 (*n* = 287); a strain expressing VPH1-GFP fusion marking the vacuole membrane in a BY4741 background (Chan and Marshall, 2014; Chan *et al.*, 2016) (VPH1-GFP, *n* = 138); and *vac14d*, a large-vacuole mutant, originally identified as a class III vacuole inheritance mutant with an enlarged and fused vacuole phenotype, involved in PI(3,5)P2 metabolism (Banta *et al.*, 1988; Bonangelino *et al.*, 2002; Rudge *et al.*, 2004; Rivero-Ríos and Weisman, 2022), in the BY4741 VPH1-GFP background, (*vac14*, *n* = 65). These measurements are based on voxel counts, scaled to isotropic voxel sizes in the range of 30 to 36 nm. A statistical summary of the sample distributions across strains showed that volume distributions and volume fractions were generally similar to previously reported values for yeast cells, vacuoles (Uchida *et al.*, 2011; Chan and Marshall, 2014), nuclei (Jorgensen *et al.*, 2007), and LDs (Uchida *et al.*, 2011; Egebjerg *et al.*, 2023) (Figure 4, statistics in Supplemental Table S3). We observed the highest variability in volume and proportional volume in vacuoles, as compared with nuclei and LDs (Figure 4, A–E). Vacuoles and nuclei had sphericity values with a median of 0.64 to 0.66 in all conditions, but vacuoles showed a higher variance in shape than did nuclei (Figure 4, F and G).

Fluorescence imaging relies on well-characterized fluorophores with specific localization and no significant impact on cellular structure or function such as viability loss or gross morphological defects. Without a way to know whether a fluorescent tag alters protein function or cellular morphology in less consequential ways, imaging of tagged strains is always associated with the potential risk that the tag itself may subtly affect organelle size or shape, particularly when, as is often the case, the proteins tagged are involved in organelle biogenesis, function, inheritance, or interactions. Due to the label-free nature of SXT, our data enable direct morphometric comparison of the parental strain and the strain expressing VPH1-GFP, a V-ATPase component protein commonly used as vacuole membrane marker for fluorescence imaging (Chan and Marshall, 2014; Chan *et al.*, 2016; Shimasawa *et al.*, 2023), allowing us to assess subtle morphological changes potentially caused by VPH1-GFP expression, despite no reported effects on cellular function. We found that VPH1-GFP expression confers small but statistically significant reductions to nuclear and LD volume distributions, but not to the volume distributions of the cell and vacuole (Figure 4, A–D). The overall proportional volume composition was very similar between VPH1-GFP and the parental strain (Figure 4E). Nuclear sphericity was significantly reduced in the VPH1-GFP strain, but there is no significant difference in vacuole sphericity (Figure 4, F and G). These results illustrate one important potential application of SXT—providing a label-free control to assess the effect of added fluorescent labels on cell structure, and the importance of characterizing controls for quantitative comparisons due to the potential presence of subtle morphological differences.

The VPH1-GFP strain, in turn, served as the direct control for the *Δvac14* strain, which also expresses VPH1-GFP. *Δvac14* is an example of a classical deletion mutant with a known morphological defect, described as “vacuolar enlargement” (Bonangelino *et al.*, 1997; Dove *et al.*, 2002; Rudge *et al.*, 2004). However, a precise quantitative description of the phenotype, such as the degree and variability of volume increase, has not been reported. Comparing VPH1-GFP and VPH1-GFP *Δvac14* showed that the mutation has only a modest effect on the average volume, but a large effect on volume variability (Figure 4B). Cells, nuclei, and LDs, were also larger in *vac14*, such that distribution (right-tailed variance) was increased for cell volume, but central tendency was increased for nuclear volume and LDs. A comparison of proportional volume composition (stacked plot of means) showed that proportional volume of the vacuole increased by 5.5% (18.9% of the cell volume in VPH1-GFP, 24.4% in *vac14*), while the “remaining cytoplasm” (a per-cell metric defined as the known organelle volume subtracted from the cell volume) was reduced by 6% (76.2% in VPH1-GFP, 70.3% in *vac14*). Volume fractions of the nucleus and LDs increased slightly (nucleus: 4.8% in VPH1-GFP, 5.5% in *vac14*; LD: 0.063% in VPH1-GFP, 0.068% in *vac14*) (Figure 4C). LDs occupy less than 0.1% of the cell volume, so the ability to detect variations within LD morphometrics are a significant strength of our approach, although such variations do not constitute significant volumetric changes for the whole cell. Vacuole sphericity was slightly increased with lower variance in *vac14* relative to VPH1-GFP, but we found no difference in nuclear sphericity. Therefore, the *Δvac14* phenotype goes beyond vacuole enlargement to include vacuole size variability, increased sphericity, and overall cell and organelle volume increase.

### High-resolution shape analysis of SXT data with meshing

Organelle shape is fundamentally linked to function. However, it is difficult to precisely quantify organelle shape in three dimensions due to limitations in throughput and resolution in most volumetric data, particularly in the *z*-axis. The high resolution and isotropy of our SXT data provide the potential for detailed 3D shape analysis of organelles, but the processing and representation of the segmented data as 3D objects are critical steps for unlocking this potential. There are multiple ways to reconstruct an organelle surface in 3D, including voxel-based reconstructions (projecting the raw image stack in three dimensions), and meshing (representing the three-dimensional [3D] surface as a simplified geometrical, typically triangulated, mesh), as exemplified in whole-cell multiorganelle visualizations for a representative cell of each strain (based on our statistical characterizations described in Figure 4) in Supplemental Figure S3 (see full rotations in Supplemental Movies S5–S10). Meshing has several advantages for shape analysis over voxel-based reconstructions. Surface information is extracted and simplified from a solid volume, reducing data size and computational burden of processing and analysis. Meshing allows the conditioning of the surface to remove jagged voxel-related artifacts, as well as customization of information density to the unique surface features of the structure, such as reducing the density of information points (triangles) in smoother regions while increasing it in feature-dense regions (Feng *et al.*, 2012; Lee *et al.*, 2020a).

Artifact removal and mesh surface conditioning also facilitate more accurate 3D morphometrics. Volume measurements taken from FIJI 3D Suite (Schindelin *et al.*, 2012; Ollion *et al.*, 2013) for voxel-based segmentations (as in Figure 4) and from GAMer2 (Lee *et al.*, 2020b) for meshes, both pre- and postmesh processing, were typically within 5% of each other (Supplemental Table S4). We note <1% volume difference between pre-and postprocessed meshes, demonstrating the volume preservation of our mesh refinement pipeline (Materials and Methods). Organelle volume ratios (vacuole:cell and nucleus:cell) were generally consistent between all measurements. However, voxel-based surface area measurements were significantly higher than those produced by mesh morphometrics, highlighting the unsuitability of voxel representations for surface area measurements due to the artifactual overestimation of surface area by voxel edges. Mesh processing had a larger effect on surface area than on volume measurements because smoothing of sharp edges and voxel terracing reduces overall surface area by 2 to 8%.

For shape quantification, sphericity measurements of voxel reconstructions and preprocessed meshes were generally well within 10%, and consistently increased after mesh processing, as expected due to smoothing, resulting in postprocessed sphericity measurements typically ∼5% higher than voxel-based measurements (Supplemental Table S4). Meshing with appropriate processing provides highly detailed surface topographies, with the potential to yield rich shape information and in-depth shape analysis beyond what is achievable with voxel-based reconstructions. To illustrate this, we demonstrate surface curvature mapping using a selection of morphologically variable vacuoles. The number of vacuoles present per cell may also be variable, ranging from 1 to 10 vacuoles per cell depending on the strain (Chan and Marshall, 2014). Clusters of several vacuoles can appear as a large mass or cluster in fluorescence imaging (Figure 1D), and in voxel reconstructions, the full detail of local and global surface features can be obscured due to occlusion of voxels and quantization artifacts (Supplemental Figure S2). Another strategy for representing surfaces that can avoid problems due to voxel artifacts is surface harmonic fitting (Marshall *et al.*, 1996; Chan and Marshall, 2014); however, this approach is limited to “star-shaped” surfaces, that is, surfaces for which every outward ray from the centroid intersects the surface at a single point. Complex organelle shapes can violate this constraint. Mesh representation is not constrained in this way and can therefore be used even for complex organelle shapes.

**FIGURE 5:**
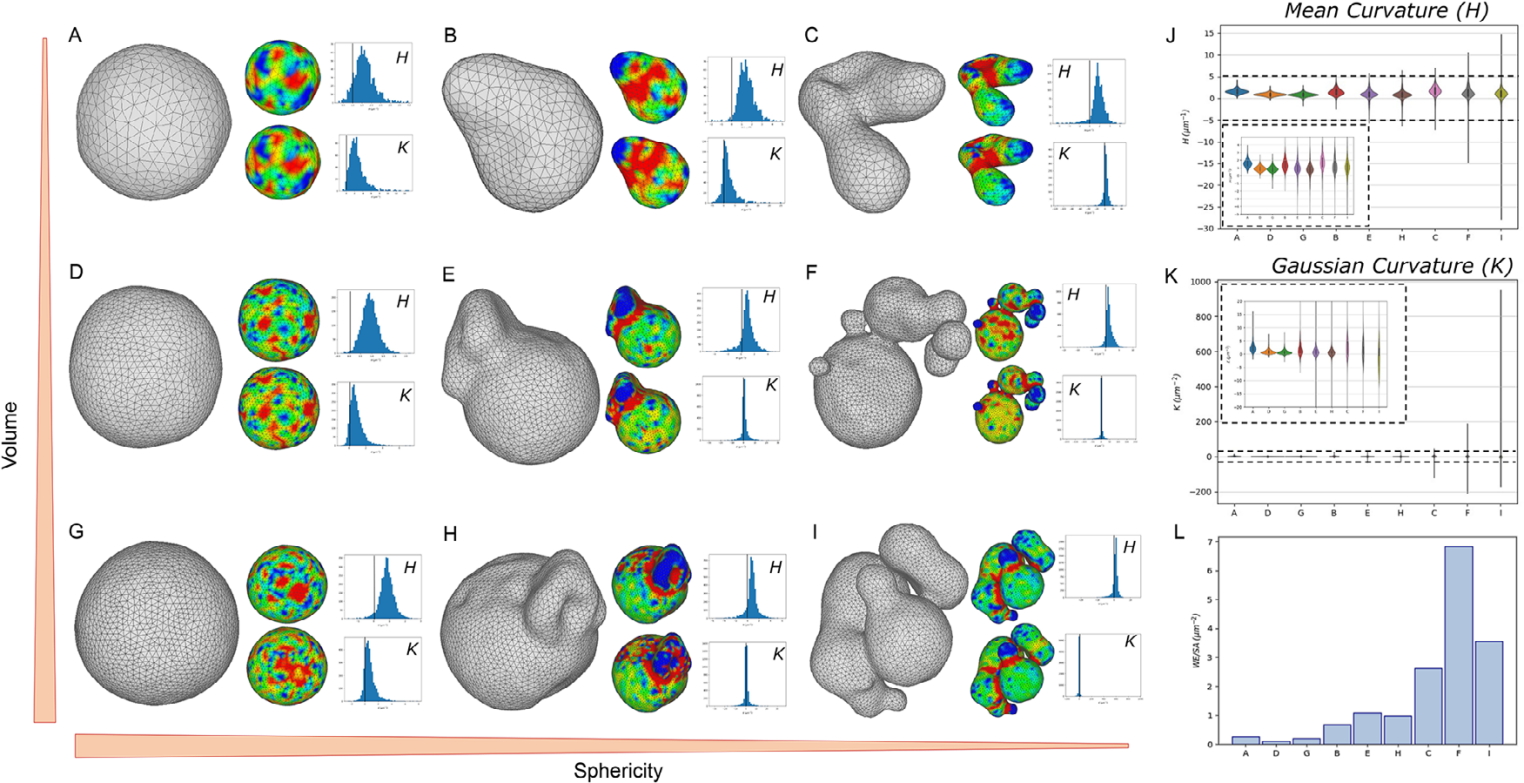
Organelle shape analysis with meshing and curvature mapping. (A–I) Example vacuole meshes presented according to increasing shape complexity (decreasing sphericity, left to right, ADG, BEH, and CFI) and increasing volume (top to bottom, ABC, DEF, and GHI). Colormaps for each vacuole display surface curvatures as mean (top, labeled *H*) and Gaussian (bottom, labeled *K*) curvature maps. Red indicates negative curvature and blue indicates positive curvature. Associated histograms show the distribution of curvatures for all vertices on the object, black vertical line indicates curvature of 0. (J and K). Mean (*H*) and Gaussian (*K*) curvature distributions plotted, grouped by volume class and ordered by shape complexity class. The main plot shows the full distributions, while insets show a *y*-axis zoom. (L) WE density (WE/SA) is calculated as described in Materials and Methods. Accompanied by Supplemental Table S5.

We asked whether meshing enables the calculation of more informative global shape metrics for complex organelles compared with crude measures obtainable from voxel reconstructions, such as sphericity. We compared several examples of vacuoles of increasing sizes and shape complexities (Figure 5; Table 1). Examples were selected to represent the observed sphericity range calculated from voxel-based segmentations (Figure 4, F and G), and volume calculated following mesh processing (Table 1). We chose small (A–C, 1.005–1.443 µm^3^), medium (E–F, 5.523–6.316 µm^3^), and large (G–I, 7.250–9.767 µm^3^) vacuoles of increasing complexity, ranging from the most sphere-like vacuoles in our dataset (ADG, 0.670–0.674), to median-range (BEH, 0.628–0.629), to the least spherical vacuoles in the dataset across all strains (CFI, 0.464–0.497). It is immediately apparent that sphericity is not adequate as a metric to distinguish objects based on shape, as the vacuoles in Figure 5, B and E have identical sphericities (0.628) but are visually different, with more pronounced protruding features in 6E.

**Table 1.** Mesh-rendered vacuole metrics accompanying Figure 5.

Vacuole	A	B	C	D	E	F	G	H	I
Volume (µm^3^)	1.005	1.443	1.304	5.802	5.523	6.316	7.25	9.767	9.65
Sphericity	0.673	0.628	0.497	0.674	0.628	0.469	0.67	0.629	0.464
Mean Curvature (avg. [SD])	1.624 (0.534)	1.45 (0.79)	1.637 (1.355)	0.915 (0.365)	0.919 (1.070)	1.199 (1.686)	0.842 (0.449)	0.766 (1.015)	0.825 (1.766)
Gaussian Curvature (avg. [SD])	2.657 (2.031)	2.04 (3.03)	1.892 (7.757)	0.846 (0.754)	0.893 (3.527)	–0.0211 (15.853)	0.722 (0.846)	0.635 (3.352)	0.254 (17.333)
SA (µm^b^)	4.876	6.411	7.664	15.718	8.175	23.291	18.195	23.418	30.858
WE	1.295	4.402	20.109	1.538	8.964	158.933	3.462	22.996	109.403
WE Density (WE/SA)	0.266	0.687	2.624	0.098	1.096	6.824	0.190	0.982	3.545

**FIGURE 6:**
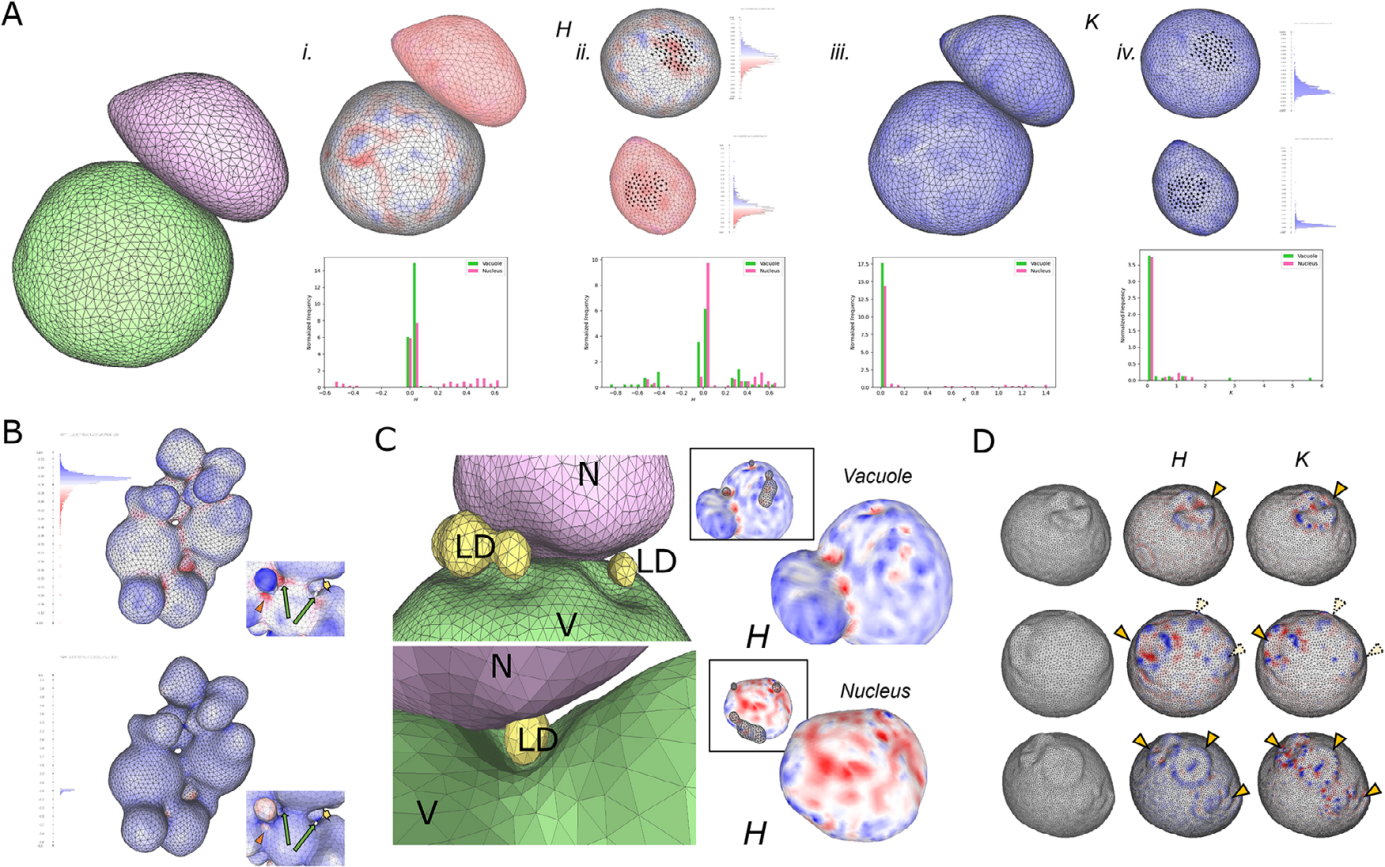
Examples of detailed organelle and cell shape features accessible in SXT data. (A) Contact interface between vacuole (green) and nucleus (pink). (i and ii) Mean (*H*) and (iii and iv) Gaussian (*K*) curvature shown as colorized surface maps and histograms for whole organelles (i and iii.) and only the interface region (ii and iv). Accompanied by Supplemental Table S7. (B) Irregularly shaped vacuole curvature maps reveal detailed surface features. Zoomed views highlight small indentations (orange arrowhead), constrictions between lobes (short yellow arrow) and internal holes (long green arrows). (C) LD (yellow) positioning near and between vacuole (green) and nucleus (pink). Mean curvature maps reveal indentations at LD sites (see inset) on vacuole surface (top) but not nucleus (bottom). (D) Bud scars on the cell wall surface. Mean curvature maps aid visualization of one (top), one clear and two putative or newly forming (middle), and three bud scars (bottom).

Curvature, a key feature of surface morphology defined as the magnitude of surface deviation from a tangent plane (Feng *et al.*, 2012), can be calculated across the mesh. Principal curvatures at each vertex (*κ*_1_ and *κ*_2_) are represented by combined measures such as mean curvature ((*κ*_1_+*κ*_2_)/2) or by Gaussian curvature (*κ*_1_*κ_2_), visualized as a color intensity gradient across the surface of the mesh. Mean curvature can be conventionally interpreted as positive for convex vertices and negative for concave vertices. Gaussian curvature can be interpreted as positive at ellipsoid or spherical regions (both principal curvatures are positive), neutral at paraboloid or cylindrical regions (*k*_2_ = 0), and negative at hyperboloid or saddle-shaped regions (Koenderink and van Doorn, 1992; Feng *et al.*, 2012). Full curvature maps for each vacuole are shown in Figure 6, A–I.

To quantitatively represent the global shape of each vacuole to distinguish between various shapes of increasing complexity using a single metric, a statistical aggregate (average and SD) of mean (*H*) or Gaussian (*K*) curvature values across the entire mesh is calculated (Figure 5, J and K; Table 1). A perfect sphere has a positive average mean curvature and Gaussian curvature, with magnitude increasing with radius, and no variance across the mesh. Increasingly complex shapes are expected to feature increasing curvature variance. As expected, as we compare vacuoles of similar sphericity with increasing volume (ADG, BEH, and CFI), mean *H* and *K* slightly decrease toward zero in central tendency (median) but increase slightly in variance. As we compare vacuoles of similar volume with increasing sphericity (ABC, DEF, and GHI), there is no meaningful change in the central tendency of *H* and *K*, but variance increases several-fold, with a dramatic increase in variance for the most complex vacuoles (CFI).

Curvature enables the calculation of Willmore energy (WE), a property related to the Helfrich energy, which, similarly to sphericity, describes the complexity of a shape as defined as its dissimilarity from a sphere (Yoon *et al.*, 2019; Mondino and Scharrer, 2020) (see Materials and Methods for full definition). We find that WE density (WE weighted by surface area) increases drastically with vacuole complexity, successfully distinguishing between shapes of similar sphericity (Figure 5L; Supplemental Table S5). Although variance of *H* and *K* and WE can all be considered reasonable metrics for overall shape quantification and comparison, with greater comparative sensitivity than sphericity, WE density is simpler to interpret. For instance, by mean and Gaussian curvature variances, vacuole I appears to be more complex than vacuole F; however, WE density, which takes both mean and Gaussian curvatures into account, shows that vacuole F is more complex (less sphere-like) than vacuole I. Overall, our curvature analysis demonstrates that the shape information provided by SXT meshes is more precise and realistic than the equivalent measured from voxel reconstructions.

As with voxel-based measurements, mesh-based morphometrics are subject to some degree of error derived from the accuracy of the segmentation. The impact of iterative autosegmentation training on mesh morphometric measurement error was measured (Supplemental Figure S4; Supplemental Table S6), using the same dataset as described earlier for voxel-based morphometrics (Supplemental Figures S1 and S2). Two representative vacuole/nucleus pairs were chosen for manual mesh processing and analysis—*vac14*-3, which showed high voxel-based measurement error for the nucleus (Supplemental Figure S4C) and low error for the vacuole (Supplemental Figure S4D); and aF-arrested cells (*aF)*, which showed low voxel-based error in both organelles. Mesh-derived vacuole and surface area measurements in the final autosegmentation (*pipe_111*) are within 10% of the manually segmented target in most cases, with the exception of the *vac14-3* nucleus which has nearly 40% error for volume (Supplemental Figure S4, A–A′) and 20% error for surface area (Supplemental Figure S4, B–B′), reflecting the error seen in voxel-based analysis. In sum, similar trends are seen in measurement error for vacuole and surface area regardless of whether the measurements are derived from voxel or mesh reconstructions. WE density calculations appear to give good agreement between target and final segmentation for vacuoles (Supplemental Figure S4, D–D′). The WED was, however, underestimated for *vac14-*3 nucleus and overestimated for the *aF* nucleus, due to a deviation in mean curvature distribution. Nevertheless, overall magnitudes of WED were within expected ranges for manual and autosegmentations each organelle, reflecting simple global shape for *vac14-3* vacuole and nucleus, and for *aF* nucleus, contrasting with a convoluted shape for the *aF* vacuole. Therefore, WED should be interpreted only as a semiquantitative measure useful for global shape comparisons.

### Examples of detailed features revealed in SXT meshes

The resolution and isotropy of our SXT data, in combination with mesh analysis, reveal detailed features of cells and organelles that are difficult to visualize otherwise, such as biologically relevant surface features, and the spatial relationships between organelles with a high level of detail. We provide several examples of such features here. The vacuole–nucleus junction (NVJ), is a metabolically sensitive signaling platform whose area fluctuates in response to starvation and other functional states (Hariri *et al.*, 2018; Pan *et al.*, 2000). We demonstrate the detection and shape characterization of the vacuole–nucleus interface region, which can be delineated using the known inter-membrane distance (IMD) range of ∼30 nm (Figure 6A; Supplemental Table S7). Although the distributions of mean and Gaussian curvatures are visibly distinct when compared across the entire vacuole and nucleus (Figure 6A, i and iii), the histograms limited to the interface region overlap (Figure 6A, ii and iv), suggesting that in those regions, the two surfaces match their curvatures at around 0, representing a flat interface. An important caveat to interface detection and analysis is that segmentation error may introduce uncertainty in IMD measurements. We found high error in minimum IMD measurements between target and autosegmented meshes in two representative cells (Supplemental Figure S4, C–C′). In the case of *vac14-3*, autosegmentation underestimates IMD relative to target, due to the overestimation of nuclear volume shown in Supplemental Figure S, 4A–A′ which reduces the space between vacuolar and nuclear surfaces. In the case of *aF*, IMD in the target and first round of segmentation training (*pipe_15)* is negative, reflecting that manual segmentation also introduces errors, in this case resulting in overlap between the organelle membranes. Iterative segmentation in fact corrects this error, resulting in IMD close to 0. From this analysis, we conclude that precise absolute measurement of IMD, and by extension, use of an absolute value of IMD to automatically detect or delineate an interface region, is not possible, given the unknown degree of error in either manual or autosegmentation, particularly when it is not possible to directly compare similar segmentation versions of the same object. Some degree of subjective judgement will be needed to identify a meaningful interface region given these considerations, and further refinement of tools and methods to precisely measure IMD and interface geometry will be needed in future work.

Figure 6B demonstrates the visibility of minute features in highly complex vacuole clusters, including small indentations, narrow constrictions, and deeply situated internal holes. The positioning of LDs situated at the periphery of the NVJ or nestled between the vacuole and nucleus (Figure 6C, top and bottom) are revealed. Mean curvature maps show that the sites of LDs correspond with indentations on the vacuole surface but not on the nucleus. Finally, cell surface geometry visualized by the mesh also reveals the location and number of bud scars, providing a physical landmark as well as potential marker of replicative age for single cells (Figure 6D). Our approach promises to reveal many more features of biological interest and enable future novel morphological analyses with high spatial precision and statistical throughput.

## DISCUSSION

We have demonstrated that SXT is unique among volumetric imaging modalities in its combination of label-free segmentation, isotropic nanoscale resolution, and capability for statistical throughput (Do *et al.*, 2015; Weinhardt *et al.*, 2019; Ekman *et al.*, 2020; Guo *et al.*, 2022; Loconte *et al.*, 2023). Historically, only optical methods, limited to ∼200 nm resolution (∼100 nm with super-resolution), offered statistically powerful sample sizes (hundreds–thousands), while higher resolution methods (EM, SXT) have been limited in throughput, especially of large volumes such as entire cells, due to laborious manual segmentation. However, the present work and other recent works (Ekman *et al.*, 2020; Egebjerg *et al.*, 2023) have demonstrated the capacity for SXT analysis to reach into sample sizes of hundreds of cells, enabling novel quantitative analyses into cellular structure. Here, we have presented a comprehensive approach to studying the subcellular structures of yeast by combining SXT, machine-learning based segmentation, statistical 3D morphometrics, and mesh surface analysis. These findings are significant as they not only provide a clear, quantitative characterization of subcellular components but also highlight the precision and utility of SXT in biological research. Our results highlight the advantages of SXT for generating high quality whole-cell, multiorganelle volumes across large sample sizes, enabling detailed comparisons between individual cells and across groups. The capability to perform statistically powered population characterization and hypothesis testing with SXT data means that questions previously only accessible by optical microscopy can now be addressed with greater resolution and structural detail, such as comparative analysis of bulk morphometrics across populations.

In the context of disease modeling, the ability to accurately measure and analyze the changes that link genetic modifications directly to phenotypic expressions at the organelle level is essential. The detailed segmentation and subsequent quantitative analysis made possible by our approach have validated its effectiveness in providing crucial insight into how the 3D morphological properties of cellular structures shift in subtle ways across perturbative conditions. Automation of image processing and analysis increases efficiency and throughput, enabling systematic data processing and analysis for otherwise prohibitively large or complex datasets. The main caveat of this is the introduction of unknown error or uncertainty. Manual validation of automated results could demand human time and effort that potentially negate the efficiency gains of the automation. This issue may be most critical when applying a trained segmentation network to a new dataset in which cells or organelles have a dramatically different morphology.

We have shown that application of the segmentation model at intermediate training stages on new cell strains (*fab1*) or conditions (aF treatment) can yield successful segmentation without incorporation of these new populations into the training set. This should be treated with the consideration, however, that the new populations are expected to be similar to the original training set. Large deviations in morphology or variability between the original and new datasets might limit the transferability of the model. Use of the model on new populations should therefore be validated first against a manually segmented subset representing the variability of the dataset. Moderately low accuracy can be remedied by incorporating a subset of the new dataset into a new round of training, while very low accuracy may require the training of a separate model more specific to the dataset to be analyzed. Ultimately, it is up to researchers to decide the degree of segmentation error that is acceptable for their purposes and to adopt an appropriate segmentation strategy accordingly.

We found that our control fluorescence strain, VPH1-GFP, exhibited slight morphological differences compared with the untagged parental strain, which were important to be aware of as a baseline for comparison with *Δvac14*. This suggests that many standardly used fluorescent protein fusion markers and strains, which are used for visualization and as controls for perturbative conditions, may themselves create subtle off-target alterations in cell structure or function. Our statistical characterization of *Δvac14* also shows that the morphological consequences of the mutant extend beyond reported characterizations such as “gross enlargement” and impaired fission of the vacuole (Bonangelino *et al.*, 1997; Rudge *et al.*, 2004; Jin *et al.*, 2008) to a more nuanced vacuole phenotype in addition to whole-cell effects. This is an example of the fact that morphological phenotypes of yeast mutants are likely far more complex than is reflected in the original qualitative characterizations from low imaging resolution in genetic screens and in concise summaries in *Saccharomyces* Genome Database (Cherry *et al.*, 1998). With the combined spatial and statistical resolution we report here, we demonstrate the potential to more thoroughly characterize and compare morphological profiles of cell types and strains than previously possible.

The unique combination of data resolution, quality, and throughput achievable through SXT autosegmentation also offers to open many novel avenues of research. SXT overcomes limitations of other tomography methods such as fixation artifacts, missing wedge artifacts and open edges thanks to a complete 180-degree tilt series and a capacity to image and reconstruct entire cellular volumes, with resolution on the scale of small suborganelle features (tens of nanometers). SXT is therefore ideally suited for surface modeling using meshing, unlocking a new avenue for precise shape analysis at scale, including surface-wide mapping and statistical characterization of curvature. Although we have shown here only a handful of examples of meshes (Figures 5 and 6; Supplemental Figure S4), the ability to first perform voxel-wise morphometrics across our entire dataset enabled us to choose statistically representative cells and pinpoint cells of interest to focus on for deeper shape analysis. In the near future, high-throughput mesh processing and analysis of hundreds or thousands of autosegmented SXT volumes with existing software such as GAMer 2 (Lee *et al.*, 2020b) or PyCurv (Salfer *et al.*, 2020) will unlock the full potential of high 3D resolution shape information with statistical power, enabling high-throughput comparative whole-cell analyses of detailed morphometrics and precise shape features of organelles and their interactions. We have described several global shape metrics that can be extracted from voxel-wise and meshed volumes, including sphericity, mean curvatures, and WE density. The exact metric can be chosen as appropriate for the given question, but ultimately such a concise shape descriptor, measured across hundreds or thousands of cells or organelles, can potentially uncover unprecedented insights into shape variability and complex features of mesoscale structures with statistical and comparative power. Such an approach promises to yield a more nuanced understanding of morphological regulation of organelles within cells, across cell populations, and between cellular contexts and genetic backgrounds. This will lead to new insights into the 3D shape, and, as statistical variation reflects dynamic range, shape dynamics of other organelles, as well as of organelle interactions and organelle networks as whole entities. The highly variable nature of 3D subcellular morphology, owing to a complex interplay of stochastic biophysical dynamics and multifaceted molecular regulatory mechanisms, will require such a statistical view to reveal meaningful new levels of understanding.

The ability to bridge biological scales by detecting fine details of organelles in a whole-cell context unlocks novel multiscale systems-level questions. What are the relationships and interdependencies between fine shape features of multiple organelles? Interorganelle membrane contact sites, which are surface regions spanning up to several microns in length (Hariri *et al.*, 2018; Uwizeye *et al.*, 2021a) defined as heterotypic organelle membranes apposed at ∼10 to 40 nm distances bridged by tethering protein complexes (Eisenberg-Bord *et al.*, 2016), are challenging to fully resolve by light microscopy. Organelle contacts have been quantified using a range of modalities and resolutions across many cellular contexts, but all approaches are subject to a tradeoff between quantitative precision, volumetric scope, and throughput. For example, it is possible to count organelle associations at the whole-cell scale from voxel colocalization in standard fluorescence data (Valm *et al.*, 2017; Shai *et al.*, 2018; Viana *et al.*, 2023), while electron microscopy enables accurate surface area and distance measurements within limited volumes (Marsh *et al.*, 2001; Crabtree *et al.*, 2024), while FIB-SEM, serial sectioning TEM, and cryo-electron tomography can achieve both, but in small sample sizes (Friedman *et al.*, 2011; Laundon *et al.*, 2019; Uwizeye *et al.*, 2021a; Uwizeye *et al.*, 2021b). Our work has made strides toward alleviating this tradeoff.

New questions surrounding global intracellular geometric patterns and organelle network arrangements become accessible with the ability to characterize cell-wide changes across cell states and perturbations with high quantitative power. What is the relationship between fine features of organelles and mesoscale cell geometry? How do organelles typically arrange in 3D space? What are the precise changes in organelle morphology, interactions, and intracellular distribution involved in complex subcellular remodeling events such as cell division, differentiation, or stress response? How are these changes linked? High-granularity cell and organelle geometries can furthermore be used as basis for structural and biophysical computational modeling, aided by better quality meshes thanks to isotropy (Lee *et al.*, 2020b; Zhu *et al.*, 2022) as well as mass density information provided by LAC. By quantifying the LAC of various organelles, and relating LAC to other cellular properties, we can gain a deeper understanding of the intrinsic biophysical properties of cells, such as density and compositional variations, which are often influenced by genetic background and cellular health. Integrating molecular, genetic, and dynamic information with the structural constraints and biophysical properties provided by SXT offers a promising avenue for holistic whole-cell modeling capable of predicting complex cell behavior (Loconte *et al.*, 2023). By bridging the submicron scale of subcellular architecture with the whole mesoscale anatomy of cells, high-throughput SXT occupies a valuable niche alongside generative 3D modeling (Murphy, 2012; Majarian *et al.*, 2019; Donovan-Maiye *et al.*, 2022), systems organelle dynamics (Mukherji and O'Shea, 2014; Wang and Mukherji, 2022), spatial proteomics (Lundberg and Borner, 2019; Cho *et al.*, 2021; Qin *et al.*, 2021; Hein *et al.*, 2023), morphological profiling (Negishi *et al.*, 2009; Chandrasekaran *et al.*, 2024; Tegtmeyer *et al.*, 2024), and other areas in the landscape of multiscale integrative cell biology, which looks toward a future holistic understanding of cellular structure and behavior ranging from molecular foundations to complex behaviors within a range of environmental contexts (Goodsell *et al.*, 2020; Rafelski and Theriot, 2024).

## MATERIALS AND METHODS

### Yeast strains and cell culture

BY4741A (haploid, mating type a), BY4741A *vph1-GFP::his3*, and BY4741A *vph1-GFP::his3 Δvac14* cells were obtained from Jennifer Fung, UCSF. BY4741A and VPH1-GFP::HIS3 have been described previously (Chan and Marshall, 2014; Chan *et al.*, 2016). The *Δvac14* strain was generated by crossing the mutant from the yeast deletion library (Huh *et al.*, 2003) with the VPH1-GFP strain. The *fab1-2* temperature-sensitive strain was a gift from Doug Koshland.

Cells were grown from frozen glycerol stocks via streaking for single colonies on YPAD agar plates with 30°C incubation. For experiments, 5-ml liquid cultures were seeded with a single plate colony and grown overnight. Fresh cultures were made using a [1:50] dilution of the stationary culture. OD_600_ was recorded immediately using a tabletop spectrophotometer and after 4 h of angled shaking or horizontal rotating incubation at 30°C. Log-phase cultures (OD_600_, 0.2–0.4) were used for confocal and SXT imaging. For *fab2-1* temperature shift, cells were grown to log-phase, followed by transfer to 37 C for 4 h. Phenotype was first validated using epifluorescence microscopy. aF arrest was performed following a previously described protocol [Diaz, 1992], overnight cultures were diluted to a concentration of 4 × 106 in 50 ml YPD media. Cultures were grown for 1 to 2 h at 24°C until reaching a density of 7 × 106, followed by addition of 7.5 µg/ml (30 µl/mL culture of 250 µg/ml stock solution in 0.1 M sodium acetate pH 5.2). After a 2-h incubation, 2.5 µg/ml (10 µl/mL the stock solution) was added to the cultures every 20 min, monitoring cell shape until at least 90% of cells display the characteristic shmoo shape, around 3 to 4 h.

### Confocal imaging and image processing

#### Live organelle staining

1mL aliquots of log-phase cell suspension were incubated with 4 µg/ml DAPI and 200 nM MitoTracker Red FM (Thermo Fisher Scientific, #M7513) at 30°C for 30 min with shaking, then centrifuged at 5000 rpm for 1 min on a tabletop centrifuge, and the pellet was resuspended in 50 to 100µL PBS. A total of 5 µl of the cell suspension were deposited on 2% agar pads, covered with a coverslip and sealed along the corners with small dabs of clear nail polish.

#### Confocal microscopy

Slides containing live cells at room temperature were imaged on a Nikon Ti inverted microscope with Yokogawa CSU-22 spinning-disk confocal, equipped with an EMCCD camera. Published images were taken using a 100x oil Plan Apo VC 1.4 objective with lateral resolution of 93 nm and axial resolution of 100 nm in the GFP (488 nm), DAPI (405 nm), and MitoTracker Red (561 nm) channels to capture entire live cells in z-stacks.

#### Image processing

Confocal images were deconvolved using Huygens software. An experimental PSF was calculated using 0.1 µm TetraSpeck Microsphere fluorescent beads (LifeTech, #T7279). Deconvolution settings are as follows: NA 1.4, RI immersion 1.52, embedding medium water 1.338 (standing in for agar pads), backprojected pinhole radius 250 nm, distance 2.53 µm. The background was manually estimated using a histogram generated in the software using a logarithmic vertical mapping function. Deconvolution was performed using an iterative classic Maximum Likelihood Estimation algorithm, using default values (40 max iterations, S:N 20, quality threshold 0.1, optimized Iteration Mode).

### Segmentation was performed individually for each channel

#### Cell

The cell boundary at the z-slice in the brightfield focal plane was manually traced and filled. This was computationally translated into a 3D volume using a custom algorithm that rotates the 2D segmentation around a central axis in 3D space, creating an ellipsoidal cell volume.

#### Vacuole

The deconvolved z-stack was processed with a 3D Gaussian Filter (Sigma 1), and Despeckle functions, followed by manual thresholding of the processed GFP signal.

#### Nucleus

The DAPI stain marked both nuclear and mitochondrial DNA. To separate the two organelle signals, Mito-tracker was also applied and imaged in a separate channel, and a custom classifier was trained using the Ilastik (Berg *et al.*, 2019) FIJI plugin. The resulting binary images were visually validated against the original fluorescence images and further cleaned with Erosion/Dilation, Fill Holes, and Despeckle processing functions.

### SXT and image processing

#### Imaging

Live cells were loaded into thin-walled cylindrical borosilicate glass capillaries (Hilgenberg GmbH, Hilgenberg, GER, catalogue no. 4023088) and then vitrified by rapidly plunging them into liquid-nitrogen–cooled (∼90 K) liquid propane. Frozen specimens were cryotransported to the soft X-ray microscope (XM-2) in the National Center for X-ray Tomography located at the Advanced Light Source of Lawrence Berkeley National Laboratory (Berkeley, CA). At XM-2, the illumination light source is produced by the 1.3 T bending magnet device and directed onto the condenser by a flat nickel mirror. The beam focused by the condenser was order-sorted by the front pinhole onto the specimen and then magnified onto the CCD detector (ANDOR, model iKon-L DO936N BN9KH, 2048 × 2048 pixels) by another micro zone plate. Projection images were collected at 517 eV using a full-rotation imaging goniometer (Le Gros *et al.*, 2014). During data collection, the cells were maintained in a stream of helium gas that had been cooled to liquid-nitrogen temperatures (Le Gros *et al.*, 2005; McDermott *et al.*, 2009). Cooling the specimen allows the collection of projection images while mitigating the effects of exposure to radiation. Each dataset (i.e., 90 projection images spanning a range of 180°) was collected using a Fresnel zone plate-based objective lens with a resolution of 50 nm. Exposure times for each projection image were in the range of 150 to 300 ms. The 2D projection images were normalized by the reference image (without a sample), automatically aligned and reconstructed in 3D volumes using the software package AREC3D (Parkinson *et al.*, 2012). The reconstructions do not require fiducial markers or manual interaction with the software and generate virtually real-time initial 3D results with the final 3D volume available within 5 min.

#### Autosegmentation

We used the model described in Ekman *et al.* (2020) for automatic segmentation, following a 3D U-Net architecture heavily inspired by nnU-Net (Isensee *et al.*, 2021). The model has a depth of five, starting with an initial filter count of 96 at the input layer. Each layer in the encoder path applies 3D convolutional operations, followed by Instance Normalization and ReLU activation. The input data consist of raw LAC volumes binned by a factor of two and processed in blocks of 96 × 96 × 96 voxels. We used the same loss function as in Isensee *et al.* (2021), which combines multilabel Cross-Entropy Loss and Dice Loss. Given the small size of the training dataset, data augmentation was applied to improve model robustness and generalization. Augmentation techniques included random intensity scaling to simulate variations in imaging conditions, along with elastic transformations to warp the input images (Çiçek *et al.*, 2016). To ensure thorough evaluation and prevent overfitting, we used a 3-fold cross-validation scheme. The dataset was evenly split into three parts, with each fold serving as a validation set during one-third of the training runs. Training progress was monitored using the validation metric, with early stopping applied when the validation loss reached its minimum.

All training was done on NVIDIA RTX A6000. Training was done with three folds using the validation set for early stopping (no meaningful change within 50 epochs). Presented training times are for 1-fold (Note: training times are approximate. They are slightly different for each fold and, because of random augmentation, not deterministic. There is ∼<10% variation between reruns.

Data | N_cells | Approx

Original | 15 | 6 h

Refinement | 53 | 30 h

Add Cells | 78 | 40 h

Final | 111 | 60 h

After training on all three folds, an ensemble approach was used for final predictions. Whole-cell images were generated by fusing an oversampled grid of block-wise label probabilities, using linear blending on overlapping volumes. The prediction probabilities from the three trained models were averaged voxel-wise to produce the final segmentation map. Our code is publicly accessible at https://github.com/ncxt/SXT_AUTOSEG.

#### Processing of segmentations

Single-cell binary segmentation images were manually examined, slight corrections to the segmentations were performed to fill in holes and remove non-specific structures, and only high quality, complete reconstructions of unbudded cells were retained in the final dataset.

Segmented individually cropped cells were manually examined against original LAC images. Cells were sorted by cell-cycle stage according to morphology (unbudded; early G_1_), budding without organelles in the bud (G_1_–early S-phase), and recently divided mother–daughter pair. Only unbudded cells were used for the morphometric analysis described here. Incomplete cells (edges cut off beyond the boundaries of the image) and poor segmentations were removed from the dataset, or, when possible, manually corrected using combinations of Erode, Dilate, and Fill Holes functions in FIJI. After quality control (QC), a few cells still had nonspecific objects segmented, such as small extra structures in the nucleus channel and tiny groups of pixels in the vacuole or LD channels that did not correspond to real organelles. Following morphometric measurements, these structures were filtered out of the dataset by setting a size threshold on the morphometric data determined for each organelle from the volume histogram for the entire population (0.3 µm^3^ for vacuoles, 0.4 µm^3^ for nuclei, and 0.02 µm^3^ for LD).

#### Voxel-based 3D morphometrics measurements and analysis

Segmented images were channel-separated such that each structure (cell, vacuole, nucleus, LD) was stored in a separate strain-specific directory. FIJI 3D Suite (Schindelin *et al.*, 2012; Ollion *et al.*, 2013) and Batch Processing was used to calculate 3D measurements in bulk using 3D Volume and 3D Surface functions. All measurements are provided in Supplemental Data File S1.

#### Mesh generation and processing

3D binary segmentation files were surface reconstructed and exported as STL files in FIJI using the 3D Viewer plugin (Schmid *et al.*, 2010), which were then processed in Blender. Each mesh was scaled by adjusting dimensions to known bounding box dimensions obtained from the FIJI's 3D Objects Counter plugin (Bolte and Cordelières, 2006). Mesh refinement was performed using the GAMer2 plugin for Blender, BlendGAMer, version 2.0.8 (Lee *et al.*, n.d.; Lee *et al.*, 2020b), using iterations of smoothing and decimation to eliminate terracing artifacts, create even triangulation, and reduce information load while preserving volume and geometry. BlendGAMer uses selective decimation, with less decimation in highly curved regions, in order to preserve complex geometries typical to high-resolution biological data. This nonuniform decimation will therefore produce inequalities in triangle size across the mesh, while optimizing triangle radius ratio. This approach was previously shown to produce meshes of sufficient quality to perform geometric analysis and finite element simulations, which are particularly sensitive to triangle aspect ratio, on highly convoluted geometries of dendritic spines and organelles from high-resolution FIB-SEM data (Lee *et al.*, 2020b).

#### Steps

A total of 15 iterations of smoothing, 10 iterations of decimation (Coarse Dense), threshold 1.5, 15 iterations of smoothing, 10 iterations of decimation (Coarse Dense), threshold 1.0, and 10 iterations of smoothing, smooth normals. The processed meshes were exported from Blender and visualized in MeshLab (Cignoni *et al.*, 2008), where a final volume-preserving Taubin smoothing step (Taubin, 1995), curvature mapping and measurement, and 3D visualization were performed. Curvature data were exported as .ply files, converted to .csv, and subsequently used for analysis.

#### WE calculation

In Figure 6, we characterize the extent of deviation from a spherical shape in terms of the WE (Yoon *et al.*, 2019), which is defined as:




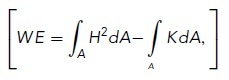




where *H* is the mean curvature, *K* is the Gaussian curvature, and *A* refers to the surface area of the vacuole. MeshLab provides the average and SD of *H* and *K*, averaged over the surface. To calculate the surface integrals from these values, we rewrite the WE as follows:




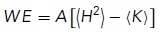




where we use the angle brackets to denote the average. We focus on the WE density, which is WE divided by the surface area, as described previously (Yoon *et al.*, 2019),




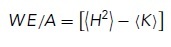




To obtain the first term on the right-hand side, we note that




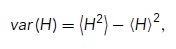




which allows us to compute the WE density in terms of the outputs from MeshLab as follows:




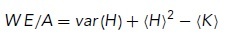




The average values of *H* and *K* are taken over mesh vertices in the software. Here, we convert these vertex-wise averages into integrals over the surface, which is strictly valid only when the mesh face areas are all equal. As a check of this assumption, we found that the integral of the Gaussian curvature calculated over the surfaces in this manner was indeed very close to 4π unless holes were present in the mesh, as required by the Gauss–Bonnet theorem (Pressley, 2010). This assumption will likely be less accurate for more highly convoluted surfaces.

#### Vacuole*–nucleus interface detection*

The Signed Distance Map function in MeshLab was used to compute the signed distance of the vacuole mesh from the nucleus reference, and vice versa, and displayed in a new layer using the Color Mapper tool. Overlapping regions on each organelle were selected using the “Conditional Face Selection” function, setting the face quality threshold as “fq<0.” The selected region was saved in a separate layer and displayed overlaid on the original curvature-mapped mesh as vertices only. Mean and Gaussian curvatures were calculated for only the interface region for each organelle and saved as .ply files for histogram plotting.

## DATA PROCESSING, ANALYSIS, AND VISUALIZATION

Morphometric and curvature data in .csv files were processed in Python Jupyter notebooks for data analysis and visualization using pandas (versions 1.3–2.2) (McKinney, 2010; The pandas development team, 2020), numpy (version 2.0) (Harris *et al.*, 2020), os, matplotlib (versions 3.5–3.9) (Hunter, 2007), and seaborn (versions 0.12–0.13) (Waskom, 2021). Analysis notebooks are publicly available on Github at https://github.com/mmirvis/SXT-autosegmentation-yeast. 3D rotation movies were taken from MeshLab via screen recording using OBSViewer (version 29.1.3) (OBS Viewer, n.d.).

## Supporting information



















Supporting Video 1 Movie S1 3D rotation of first example cell (top row Figure 1D), reconstructed by confocal microscopy.

Supporting Video 2 Movie S2 3D rotation of second example cell (second row Figure 1D), reconstructed by confocal microscopy.

Supporting Video 3 Movie S3 3D rotation of third example cell (third row Figure 1D), reconstructed by SXT.

Supporting Video 4 Movie S4 3D rotation of fourth example cell (fourth row Figure 1D), reconstructed by SXT.

Supporting Video 5 Movie S5 3D rotation of voxel based reconstruction of WT cell as depicted in Supplemental Figure S3A.

Supporting Video 6 Movie S6 3D rotation of voxel based reconstruction of VPH1-GFP cell as depicted in Supplemental Figure S3B.

Supporting Video 7 Movie S7 3D rotation of voxel based reconstruction of vac14 cell as depicted in Supplemental Figure S3C.

Supporting Video 8 Movie S8 3D rotation of surface mesh reconstruction of WT cell as depicted in Supplemental Figure S3D.

Supporting Video 9 Movie S9 3D rotation of surface mesh reconstruction of VPH1-GFP cell as depicted in Supplemental Figure S3E.

Supporting Video 10 Movie S10 3D rotation of surface mesh reconstruction of vac14 cell as depicted in Supplemental Figure S3F.

## Abbreviations used:

3D: three-dimensional
CNN: convolutional neural network
EM: electron microscopy
FIJI: FIJI Is Just ImageJ
GAMer: Geometry-preserving Adaptive MeshER software
GFP: green fluorescent protein
LAC: linear absorption coefficient
SXT: soft X-ray tomography
WE: Willmore energy
WED: Willmore energy density.
