# Supplementary material for: Automated segmentation of soft X-ray tomography: Native cellular structure with submicron resolution at high-throughput for whole-cell quantitative imaging in yeast
Source: Mol Biol Cell. 2025 Oct 1;36(10):ar132. doi: 10.1091/mbc.E24-10-0486 (PMC12509287; doi:10.1091/mbc.E24-10-0486)
Supplement: Supplementary file 1 [file mbc-36-ar132-s001.pdf]

Supplemental Materials

Molecular Biology of the Cell

Chen *et al*.

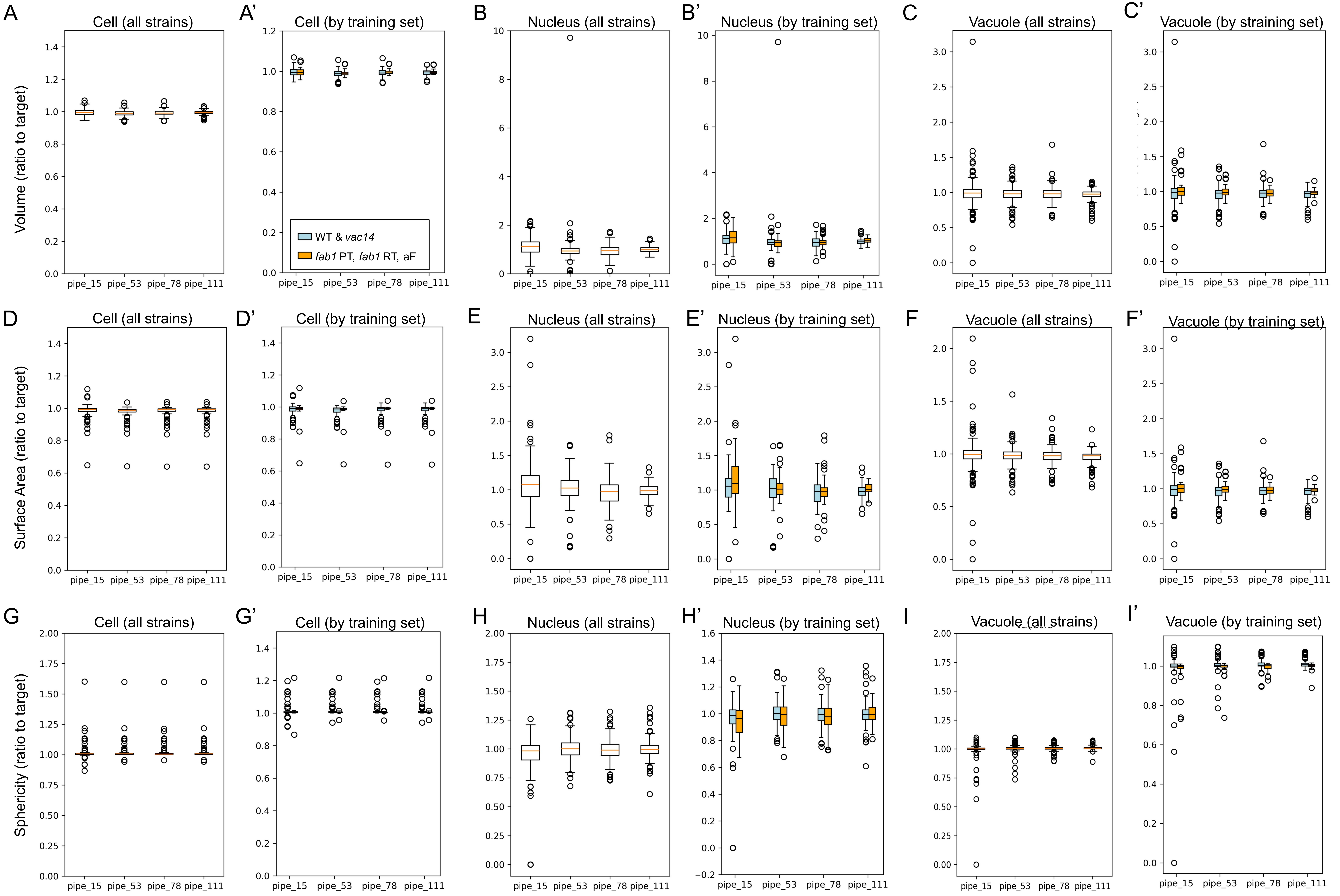
A
Cell (all strains)
A'
Cell (by training set)
B
Nucleus (all strains)
B'
Nucleus (by training set)
C
Vacuole (all strains)
C'
Vacuole (by straining set)
Volume (ratio to target)
WT & vac14
fab1 PT, fab1 RT, aF
D
Cell (all strains)
D'
Cell (by training set)
E
Nucleus (all strains)
E'
Nucleus (by training set)
F
Vacuole (all strains)
F'
Vacuole (by training set)
Surface Area (ratio to target)
G
Cell (all strains)
G'
Cell (by training set)
H
Nucleus (all strains)
H'
Nucleus (by training set)
I
Vacuole (all strains)
I'
Vacuole (by training set)
Sphericity (ratio to target)
pipe_15
pipe_53
pipe_78
pipe_111

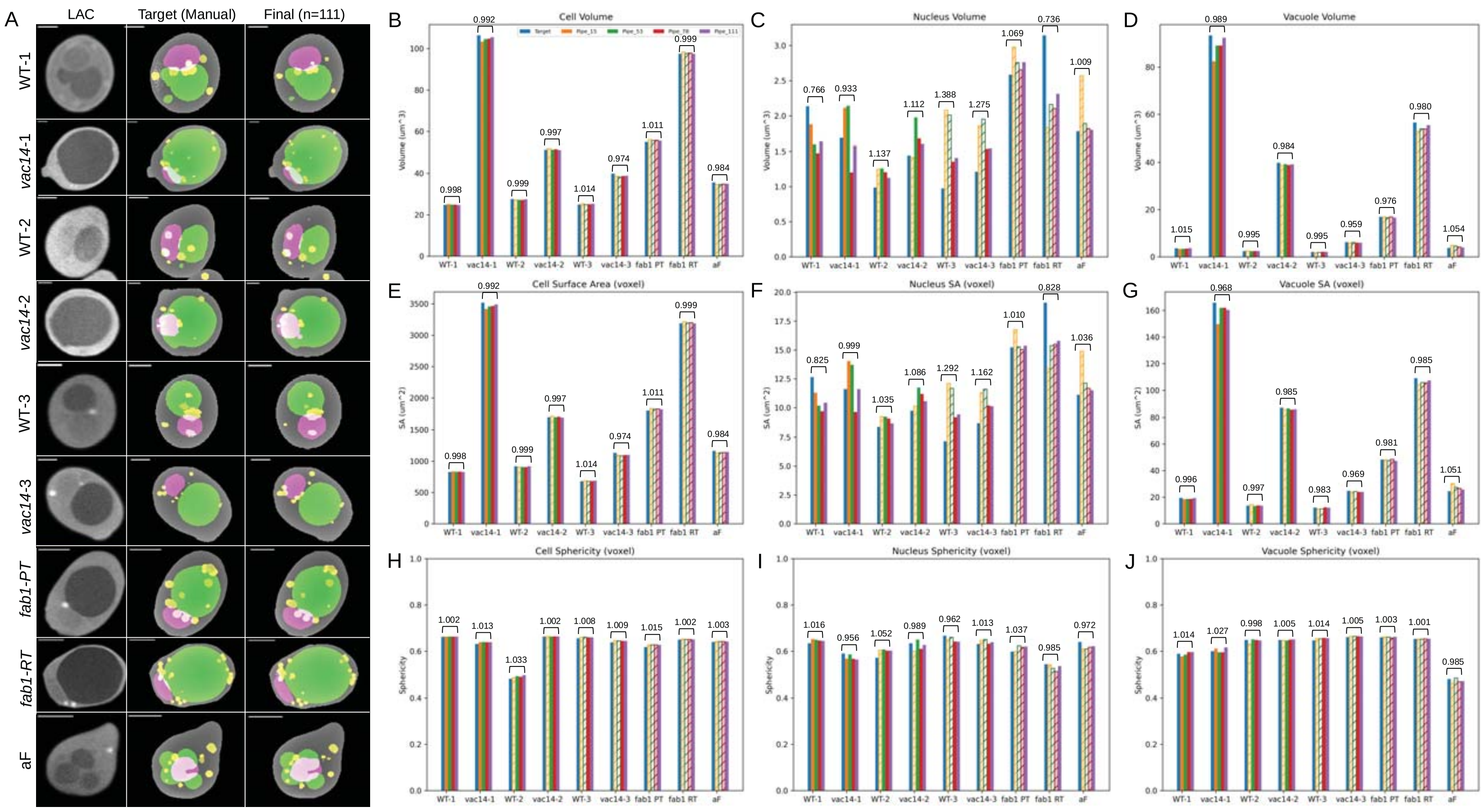
A
LAC
Target (Manual)
Final (n=111)
WT-1
vac14-1
WT-2
vac14-2
WT-3
vac14-3
fab1-PT
fab1-RT
aF
B
Cell Volume
C
Nucleus Volume
D
Vacuole Volume
E
Cell Surface Area (voxel)
F
Nucleus SA (voxel)
G
Vacuole SA (voxel)
H
Cell Sphericity (voxel)
I
Nucleus Sphericity (voxel)
J
Vacuole Sphericity (voxel)

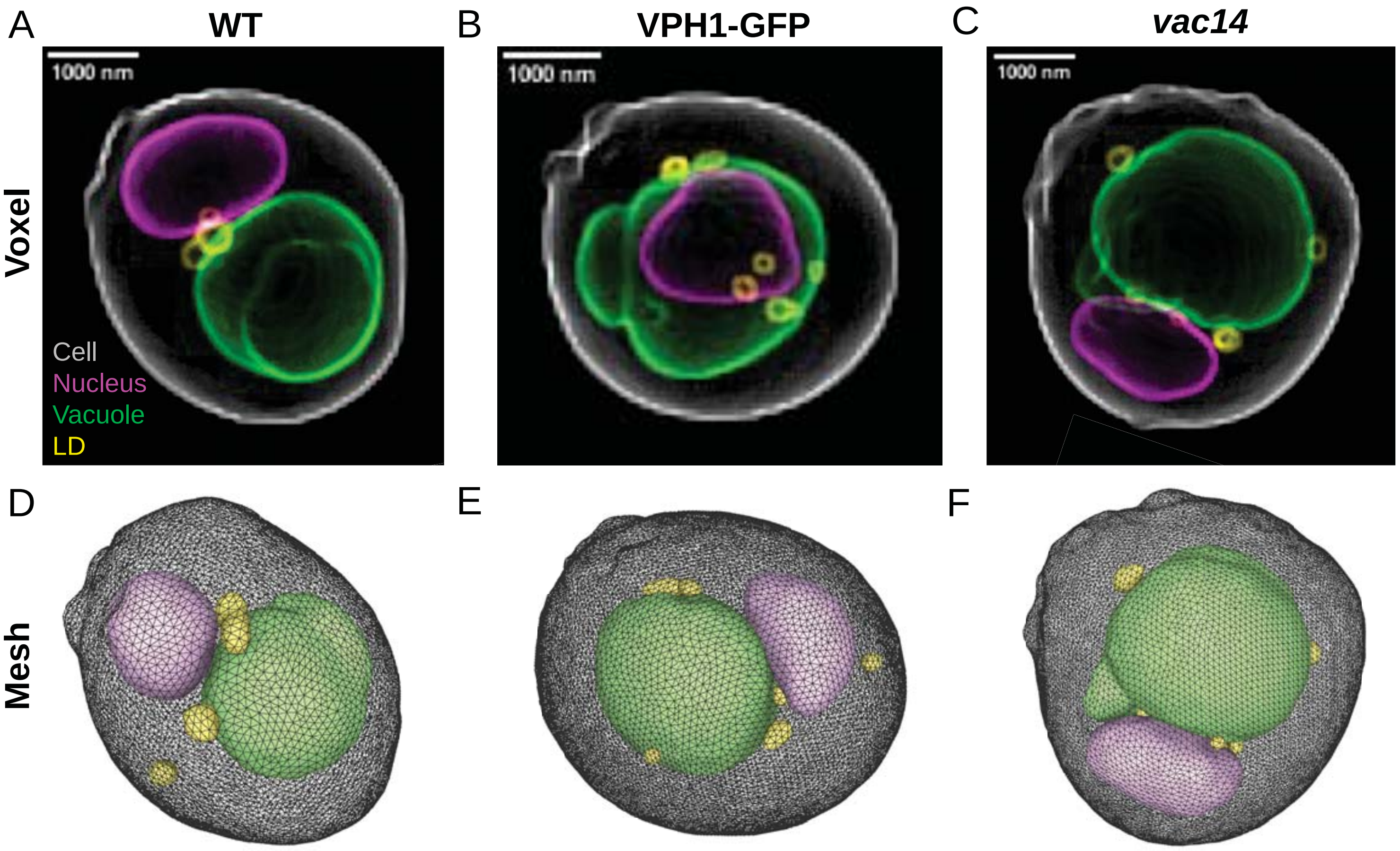
A
WT
B
VPH1-GFP
C
vac14
1000 nm
1000 nm
1000 nm
Voxel
Cell
Nucleus
Vacuole
LD
D
E
F
Mesh

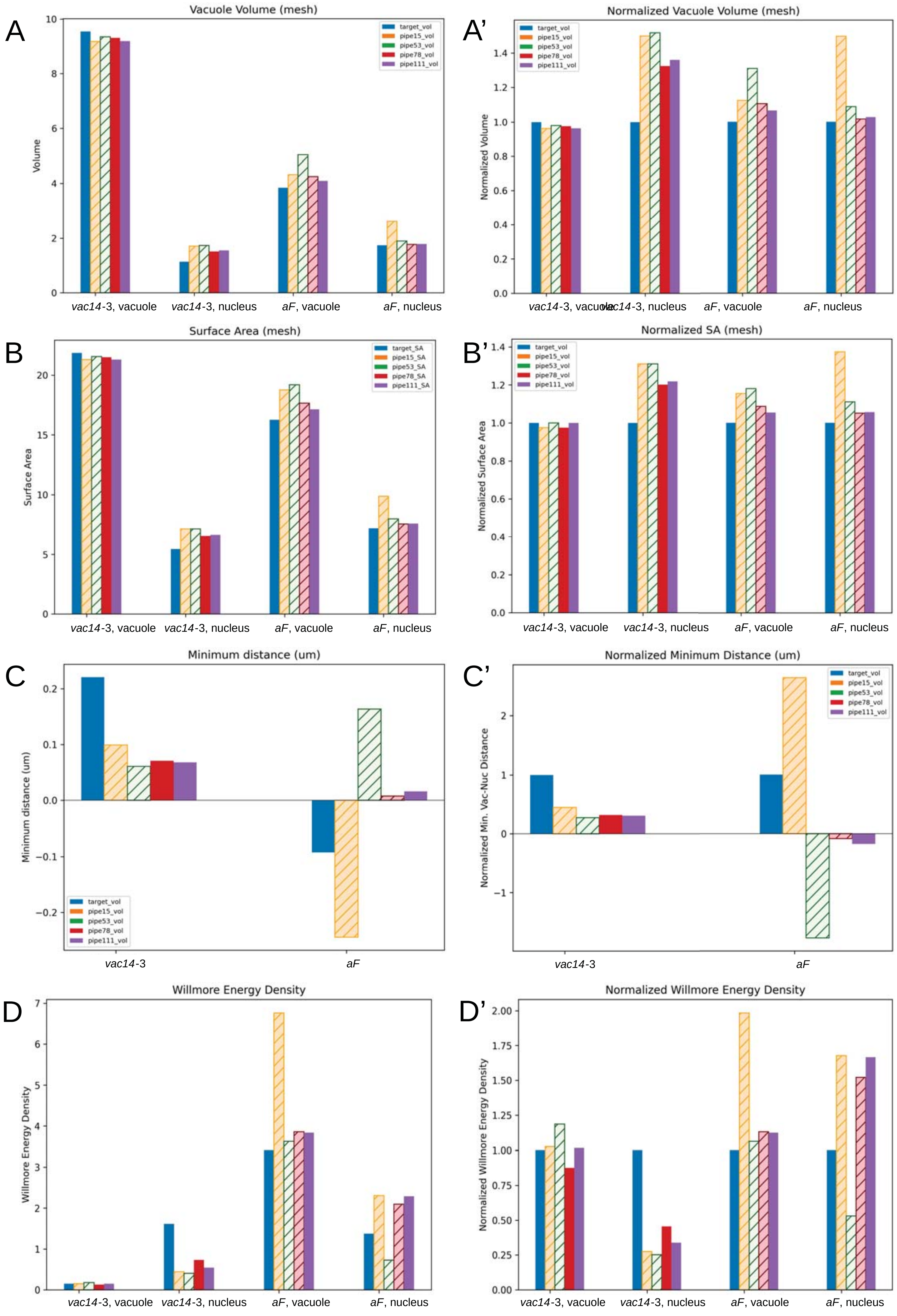
A
Vacuole Volume (mesh)
Volume
target_vol
pipe15_vol
pipe53_vol
pipe78_vol
pipe111_vol
vac14-3, vacuole
vac14-3, nucleus
aF, vacuole
aF, nucleus
A'
Normalized Vacuole Volume (mesh)
Normalized Volume
target_vol
pipe15_vol
pipe53_vol
pipe78_vol
pipe111_vol
vac14-3, vacuole
vac14-3, nucleus
aF, vacuole
aF, nucleus
B
Surface Area (mesh)
Surface Area
target_SA
pipe15_SA
pipe53_SA
pipe78_SA
pipe111_SA
vac14-3, vacuole
vac14-3, nucleus
aF, vacuole
aF, nucleus
B'
Normalized SA (mesh)
Normalized Surface Area
target_vol
pipe15_vol
pipe53_vol
pipe78_vol
pipe111_vol
vac14-3, vacuole
vac14-3, nucleus
aF, vacuole
aF, nucleus
C
Minimum distance (um)
Minimum distance (um)
target_vol
pipe15_vol
pipe53_vol
pipe78_vol
pipe111_vol
vac14-3
aF
C'
Normalized Minimum Distance (um)
Normalized Min. Vac-Nuc Distance
target_vol
pipe15_vol
pipe53_vol
pipe78_vol
pipe111_vol
vac14-3
aF
D
Willmore Energy Density
Willmore Energy Density
vac14-3, vacuole
vac14-3, nucleus
aF, vacuole
aF, nucleus
D'
Normalized Willmore Energy Density
Normalized Willmore Energy Density
vac14-3, vacuole
vac14-3, nucleus
aF, vacuole
aF, nucleus

Figure S1. **Iterative autosegmentation model training improves voxel-based morphometric measurements relative to manually segmented reference.** Voxel-based (A-C) volume, (D-F) surface area, and (G-I) sphericity were calculated for all segmentations for each (ADG) cell, (BEH) nucleus, and (CFI) vacuole segmentation. Box-and-whisker plots represent the distribution of auto-segmentation errors relative to the manual segmentation calculated as voxel auto-to-manual ratio (“ratio to target”). A’-I’) The same data as in A-I, split by strains. Light blue boxes correspond to WT and *vac14*, which were used in the first 3 rounds of training (*pipe_15, pipe53, pipe_78)*. Orange boxes correspond to *fab1* PT, *fab1* RT, and alpha-factor arrested cells (aF), which were added in the final round of training (*pipe_111)*, demonstrating the generalizability of the model to new datasets. G’ inset shows a y-axis zoom to 0.8-1.3. Accompanied by Table S1.

Figure S2. Voxel metric evolution over successive rounds of model training for 9 representative cells. A) LAC and 3D projections of manual (”target”) and final auto-segmentation (“pipe_111”). Green objects represent vacuoles, magenta objects represent nuclei, and yellow objects represent lipid droplets. Scale bar = 1 µm. Voxel-based B-D) volume, E-G) surface area, and H-J) sphericity were measured for each set of BEH) cell, CFI) nucleus, and DGJ) vacuole segmentations. The order of cells on the x-axis from left to right corresponds to the order of cells, top to bottom, in panel A. Hatched bars indicate segmentation versions in which the cell was not included in the training set, while solid bars indicate that the cell was included. WT-1 and vac14-1 were included in the initial training set (pipe_15), WT-2 and vac14-2 were added in pipe_53,

WT-3 and vac14-3 were added in pipe_78, and fab1 PT, fab1 RT, and alpha-factor were added in the final round (pipe_111). Values above each cell indicate ratio of final segmentation (pipe_111) relative to manually segmented target. Accompanied by Table S2.

Figure S3. Whole-cell voxel-based and mesh renderings of statistical representatives from each strain. (A-C) Voxel-based reconstructions and (D-F) refined mesh renderings of whole cells including organelles are shown for statistically representative cells from (A,D) WT, (B,E) VPH1-GFP, and (C,F) *vac14* strains, including nucleus (magenta/pink), vacuole (green), lipid droplets (yellow, SXT only), and cell wall (white/gray). See corresponding rotation movies in Supplementary Movies 5-10. Measurements provided in Table S4.

Figure S4. Mesh-derived morphometric evolution over successive rounds of model training. Vacuole-nucleus pairs are analyzed from two representative cells, one with high segmentation error for nucleus (vac14-3) and one with low segmentation error (aF). Mesh-based A, A') volume, B, B') surface area, and C, C') minimum vacuole-nucleus inter-membrane distance, and D, D') Willmore energy density were calculated for each organelle. Raw values (ABCD) as well as values normalized to target segmentation (A'B'C'D') are shown in order to display the error for each organelle metric. As in Figure S3, hatched bars indicate segmentation versions in which the cell was not included in the training set, while solid bars indicate that the cell was included. Accompanied by Table S6.

ID_x	Cell ID	Pipe introduced
cell_target_2_multi_bin	BY471A_1208_1_10_0	15
cell_pipe_15_predict_2_multi_bin	BY471A_1208_1_10_0	15
cell_pipe_53_predict_2_multi_bin	BY471A_1208_1_10_0	15
cell_pipe_78_predict_2_multi_bin	BY471A_1208_1_10_0	15
cell_pipe_111_predict_2_multi_bin	BY471A_1208_1_10_0	15
cell_target_62_multi_bin	vac14_1361_4_9	15
cell_pipe_15_predict_62_multi_bin	vac14_1361_4_9	15
cell_pipe_53_predict_62_multi_bin	vac14_1361_4_9	15
cell_pipe_78_predict_62_multi_bin	vac14_1361_4_9	15
cell_pipe_111_predict_62_multi_bin	vac14_1361_4_9	15
cell_target_7_multi_bin	BY471A_1208_1_3_0	53
cell_pipe_15_predict_7_multi_bin	BY471A_1208_1_3_0	53
cell_pipe_53_predict_7_multi_bin	BY471A_1208_1_3_0	53
cell_pipe_78_predict_7_multi_bin	BY471A_1208_1_3_0	53
cell_pipe_111_predict_7_multi_bin	BY471A_1208_1_3_0	53
cell_target_46_multi_bin	vac14_1361_20_3	53
cell_pipe_15_predict_46_multi_bin	vac14_1361_20_3	53
cell_pipe_53_predict_46_multi_bin	vac14_1361_20_3	53
cell_pipe_78_predict_46_multi_bin	vac14_1361_20_3	53
cell_pipe_111_predict_46_multi_bin	vac14_1361_20_3	53
cell_target_100_multi_bin	wt_BY4741A_1463_31_14	78
cell_pipe_15_predict_100_multi_bin	wt_BY4741A_1463_31_14	78
cell_pipe_53_predict_100_multi_bin	wt_BY4741A_1463_31_14	78
cell_pipe_78_predict_100_multi_bin	wt_BY4741A_1463_31_14	78
cell_pipe_111_predict_100_multi_bin	wt_BY4741A_1463_31_14	78
cell_target_75_multi_bin	vphGFP_VAC14_1464_22_V0_1011_1	78
cell_pipe_15_predict_75_multi_bin	vphGFP_VAC14_1464_22_V0_1011_1	78
cell_pipe_53_predict_75_multi_bin	vphGFP_VAC14_1464_22_V0_1011_1	78
cell_pipe_78_predict_75_multi_bin	vphGFP_VAC14_1464_22_V0_1011_1	78
cell_pipe_111_predict_75_multi_bin	vphGFP_VAC14_1464_22_V0_1011_1	78
cell_target_35_multi_bin	FAB1_RT_PT_5190_10_0	111
cell_pipe_15_predict_35_multi_bin	FAB1_RT_PT_5190_10_0	111
cell_pipe_53_predict_35_multi_bin	FAB1_RT_PT_5190_10_0	111
cell_pipe_78_predict_35_multi_bin	FAB1_RT_PT_5190_10_0	111
cell_pipe_111_predict_35_multi_bin	FAB1_RT_PT_5190_10_0	111
cell_target_30_multi_bin	FAB1_RT_37_9665_4_3	111
cell_pipe_15_predict_30_multi_bin	FAB1_RT_37_9665_4_3	111
cell_pipe_53_predict_30_multi_bin	FAB1_RT_37_9665_4_3	111
cell_pipe_78_predict_30_multi_bin	FAB1_RT_37_9665_4_3	111
cell_pipe_111_predict_30_multi_bin	FAB1_RT_37_9665_4_3	111
cell_target_82_multi_bin	alphaF_plus_5178_10_10	111
cell_pipe_15_predict_82_multi_bin	alphaF_plus_5178_10_10	111
cell_pipe_53_predict_82_multi_bin	alphaF_plus_5178_10_10	111
cell_pipe_78_predict_82_multi_bin	alphaF_plus_5178_10_10	111
cell_pipe_111_predict_82_multi_bin	alphaF_plus_5178_10_10	111

Fig S2

Object ID	Strain	Pix size	Cell Volume
cell_target_2_multi_bin.tiff_1	WT	0.03	24.77628
cell_pipe_15_predict_2_multi_bin.tiff_1	WT	0.03	25.108083
cell_pipe_53_predict_2_multi_bin.tiff_1	WT	0.03	24.894756
cell_pipe_78_predict_2_multi_bin.tiff_1	WT	0.03	24.899508
cell_pipe_111_predict_2_multi_bin.tiff_1	WT	0.03	24.73173
cell_target_62_multi_bin.tiff_1	vac14	0.03023	106.3241143
cell_pipe_15_predict_62_multi_bin.tiff_1	vac14	0.03023	103.2810249
cell_pipe_53_predict_62_multi_bin.tiff_1	vac14	0.03023	104.4950395
cell_pipe_78_predict_62_multi_bin.tiff_1	vac14	0.03023	104.7312675
cell_pipe_111_predict_62_multi_bin.tiff_1	vac14	0.03023	105.513657
cell_target_7_multi_bin.tiff_1	WT	0.03	27.529227
cell_pipe_15_predict_7_multi_bin.tiff_1	WT	0.03	27.141399
cell_pipe_53_predict_7_multi_bin.tiff_1	WT	0.03	27.228366
cell_pipe_78_predict_7_multi_bin.tiff_1	WT	0.03	27.143667
cell_pipe_111_predict_7_multi_bin.tiff_1	WT	0.03	27.520128
cell_target_46_multi_bin.tiff_1	vac14	0.03023	51.33691902
cell_pipe_15_predict_46_multi_bin.tiff_1	vac14	0.03023	51.86288612
cell_pipe_53_predict_46_multi_bin.tiff_1	vac14	0.03023	51.37484921
cell_pipe_78_predict_46_multi_bin.tiff_1	vac14	0.03023	51.6264095
cell_pipe_111_predict_46_multi_bin.tiff_1	vac14	0.03023	51.18555741
cell_target_100_multi_bin.tiff_1	WT	0.0368	24.94158844
cell_pipe_15_predict_100_multi_bin.tiff_1	WT	0.0368	25.28112133
cell_pipe_53_predict_100_multi_bin.tiff_1	WT	0.0368	24.8669839
cell_pipe_78_predict_100_multi_bin.tiff_1	WT	0.0368	25.05636082
cell_pipe_111_predict_100_multi_bin.tiff_1	WT	0.0368	25.29667017
cell_target_75_multi_bin.tiff_1	vac14	0.03531	39.90029936
cell_pipe_15_predict_75_multi_bin.tiff_1	vac14	0.03531	38.64617712
cell_pipe_53_predict_75_multi_bin.tiff_1	vac14	0.03531	38.23358072
cell_pipe_78_predict_75_multi_bin.tiff_1	vac14	0.03531	38.74708098
cell_pipe_111_predict_75_multi_bin.tiff_1	vac14	0.03531	38.85309166
cell_target_35_multi_bin.tiff_1	fab1 PT	0.030572	54.92495045
cell_pipe_15_predict_35_multi_bin.tiff_1	fab1 PT	0.030572	56.04362387
cell_pipe_53_predict_35_multi_bin.tiff_1	fab1 PT	0.030572	55.58778231
cell_pipe_78_predict_35_multi_bin.tiff_1	fab1 PT	0.030572	55.76877024
cell_pipe_111_predict_35_multi_bin.tiff_1	fab1 PT	0.030572	55.55240765
cell_target_30_multi_bin.tiff_1	fab1 RT	0.030572	97.51177601
cell_pipe_15_predict_30_multi_bin.tiff_1	fab1 RT	0.030572	98.31004879
cell_pipe_53_predict_30_multi_bin.tiff_1	fab1 RT	0.030572	97.58403974
cell_pipe_78_predict_30_multi_bin.tiff_1	fab1 RT	0.030572	97.80080236
cell_pipe_111_predict_30_multi_bin.tiff_1	fab1 RT	0.030572	97.42839698
cell_target_82_multi_bin.tiff_1	WT aF	0.030572	35.57230032
cell_pipe_15_predict_82_multi_bin.tiff_1	WT aF	0.030572	34.57106618
cell_pipe_53_predict_82_multi_bin.tiff_1	WT aF	0.030572	34.67530426
cell_pipe_78_predict_82_multi_bin.tiff_1	WT aF	0.030572	34.78168538
cell_pipe_111_predict_82_multi_bin.tiff_1	WT aF	0.030572	35.0065916

2B	Fig S2E		Fig S2H		Fig S2C	
**Cell Vol Error**	**Cell SA**	**Cell SA Error**	**Cell Sphericity**	**Cell Spher Error**	**Nuc Volume**	**Nuc Vol Error**
1	825.876	1	0.662	1	2.136942	1
1.013391962	836.9361	1.01339196	0.663	1.001510574	1.8792	0.87938746
1.004781832	829.8252	1.00478183	0.663	1.001510574	1.594485	0.74615268
1.004973628	829.9836	1.00497363	0.663	1.001510574	1.472985	0.68929573
**0.998201909**	824.391	**0.99820191**	0.663	**1.001510574**	1.636902	**0.76600207**
1	3517.17216	1	0.631	1	1.68942653	1
0.971379123	3416.50761	0.97137912	0.639	1.012678288	2.11370316	1.25113648
0.982797178	3456.66687	0.98279718	0.64	1.014263074	2.14141181	1.26753769
0.98501895	3464.48123	0.98501895	0.639	1.012678288	1.20125149	0.71104098
**0.992377484**	3490.36246	**0.99237748**	0.639	**1.012678288**	1.57696201	**0.93343036**
1	917.6409	1	0.481	1	0.98847	1
0.985912136	904.7133	0.98591214	0.485	1.008316008	1.248318	1.26287899
0.989071215	907.6122	0.98907122	0.492	1.022869023	1.257363	1.2720295
0.985994521	904.7889	0.98599452	0.489	1.016632017	1.204983	1.21903851
**0.999669479**	917.3376	**0.99966948**	0.497	**1.033264033**	1.123794	**1.13690249**
1	1698.21102	1	0.663	1	1.44123659	1
1.010245397	1715.60986	1.0102454	0.664	1.001508296	1.41247816	0.980046
1.000738848	1699.46574	1.00073885	0.663	1	1.97841975	1.37272379
1.005639031	1707.78728	1.00563903	0.664	1.001508296	1.67774083	1.16409814
**0.997051603**	1693.20402	**0.9970516**	0.664	**1.001508296**	1.60223959	**1.11171171**
1	677.760556	1	0.655	1	0.97474295	1
1.013613122	686.986993	1.01361312	0.66	1.007633588	1.40741938	1.44388772
0.99700883	675.733258	0.99700883	0.661	1.009160305	2.07955794	2.13344241
1.004601647	680.87937	1.00460165	0.66	1.007633588	2.01043537	2.06252876
**1.014236532**	687.409516	**1.01423653**	0.66	**1.007633588**	1.35334728	**1.38841454**
1	1129.99998	1	0.638	1	1.21229908	1
0.968568601	1094.4825	0.9685686	0.645	1.010971787	1.85743221	1.53215673
0.958227917	1082.79753	0.95822792	0.644	1.009404389	1.95133619	1.60961615
0.971097501	1097.34016	0.9710975	0.644	1.009404389	1.53609833	1.26709518
**0.97375439**	1100.34244	**0.97375439**	0.644	**1.009404389**	1.54551954	**1.27486654**
1	1796.57695	1	0.618	1	2.58506426	1
1.020367309	1833.16839	1.02036731	0.627	1.014563107	2.97587131	1.15117886
1.012067956	1818.25796	1.01206796	0.627	1.014563107	2.75879438	1.06720534
1.015363142	1824.17801	1.01536314	0.627	1.014563107	2.65407055	1.02669423
**1.011423901**	1817.10087	**1.0114239**	0.627	**1.014563107**	2.76382341	**1.06915076**
1	3189.57791	1	0.65	1	3.14311513	1
1.008186424	3215.68915	1.00818642	0.652	1.003076923	1.83556735	0.58399622
1.000741077	3191.94164	1.00074108	0.651	1.001538462	2.16328294	0.6882608
1.002964015	3199.03187	1.00296401	0.651	1.001538462	2.10347749	0.66923336
**0.999144934**	3186.85061	**0.99914493**	0.651	**1.001538462**	2.31278229	**0.73582487**
1	1163.55817	1	0.64	1	1.78121954	1
0.971853545	1130.80813	0.97185355	0.642	1.003125	2.56869133	1.44209699
0.974783861	1134.21772	0.97478386	0.642	1.003125	1.89022948	1.06119961
0.977774422	1137.69742	0.97777442	0.642	1.003125	1.81833721	1.02083835
**0.984096932**	1145.05402	**0.98409693**	0.642	**1.003125**	1.79810679	**1.00948073**

Fig S2F		Fig S2I		Fig S2D		Fig S
**Nuc SA**	**Nuc SA Error**	**Nuc Sphericity**	**Nuc Spher Error**	**Vac Volume**	**Vac Vol Error**	**Vac SA**
12.6486	1	0.634	1	3.650697	1	19.4652
11.295	0.8929842	0.652	1.028391167	3.289221	0.900984387	18.5256
10.1772	0.80461079	0.649	1.023659306	3.405051	0.932712575	18.7002
9.6894	0.76604525	0.646	1.018927445	3.503061	0.959559503	18.7074
10.4292	**0.82453394**	0.644	**1.015772871**	3.705264	**1.014947009**	19.3824
11.6187258	1	0.59	1	93.32708648	1	165.806426
14.0349528	1.20795973	0.568	0.962711864	82.44316154	0.883378713	149.815019
13.7023104	1.17932987	0.586	0.993220339	89.053905	0.954212848	161.986243
9.63018186	0.82885009	0.567	0.961016949	89.0927345	0.954628906	162.093467
11.6095872	**0.99921347**	0.564	**0.955932203**	92.33411681	**0.989360327**	160.472632
8.3844	1	0.572	1	2.478777749	1	13.6748948
9.288	1.10777158	0.604	1.055944056	2.576628238	1.039475297	14.4224265
9.2592	1.10433663	0.608	1.062937063	2.406563978	0.970867186	13.3331138
9.1026	1.08565908	0.602	1.052447552	2.510492137	1.012794365	13.7772463
8.6814	**1.03542293**	0.602	**1.052447552**	2.466732912	**0.995140816**	13.636513
9.73070568	1	0.634	1	39.76955222	1	86.9394995
10.1401118	1.04207363	0.6	0.94637224	38.5908304	0.970361199	85.4010829
11.7338712	1.20586026	0.65	1.025236593	39.21273424	0.985998887	86.3436339
11.1892149	1.1498873	0.61	0.96214511	38.84035941	0.976635573	85.31712
10.5659672	**1.08583772**	0.627	**0.988958991**	39.11505562	**0.983542772**	85.6231782
7.13142784	1	0.667	1	2.071760314	1	12.1504134
9.46884608	1.32776301	0.641	0.96101949	1.839196254	0.887745673	11.1036085
12.0987802	1.69654387	0.651	0.976011994	1.869170415	0.90221364	11.2325899
11.6789658	1.63767566	0.66	0.989505247	2.194171474	1.05908558	12.4065067
9.21695744	**1.29244208**	0.642	**0.962518741**	2.060902181	**0.994758982**	11.9391831
8.70762396	1	0.631	1	6.226996296	1	24.7457188
11.2934791	1.29696449	0.647	1.025356577	5.928911976	0.95213032	23.8652812
11.5976973	1.33190149	0.651	1.031695721	6.084697608	0.977148101	24.2615716
10.1938049	1.17067583	0.632	1.001584786	6.005518961	0.964432718	24.0316484
10.1214907	**1.16237113**	0.639	**1.012678288**	5.974458986	**0.959444763**	23.9886546
15.2235333	1	0.598	1	16.92440504	1	48.2806764
16.7451389	1.09995088	0.598	1	16.90564714	0.998891666	48.1362877
15.2777429	1.0035609	0.623	1.04180602	16.52184227	0.976214067	47.4947629
15.0384732	0.98784381	0.616	1.030100334	16.88724838	0.997804551	48.4506531
15.3712076	**1.00970039**	0.62	**1.036789298**	16.51960459	**0.97608185**	47.3631681
19.0836262	1	0.544	1	56.585655	1	109.0908
13.4047099	0.70241943	0.541	0.994485294	52.824744	0.933535964	104.3532
15.3730769	0.80556372	0.526	0.966911765	53.880309	0.95219025	105.7428
15.494581	0.81193065	0.512	0.941176471	53.781435	0.950442917	105.561
15.7917988	**0.82750514**	0.536	**0.985294118**	55.432161	**0.979615081**	107.4258
11.1073471	1	0.64	1	3.79954641	1	24.5326193
14.9057533	1.3419724	0.609	0.9515625	4.917276887	1.294174713	30.1554567
12.135459	1.09256143	0.609	0.9515625	4.516897527	1.188799146	27.299175
11.671874	1.05082464	0.617	0.9640625	4.146263735	1.091252294	26.5589344
11.5055068	**1.03584652**	0.622	**0.971875**	4.005308027	**1.054154258**	25.7757

S2G	Fig S2J	
**Vac SA Error**	**Vac Sphericity**	**Vac Spher Error**
1	0.589	1
0.95172924	0.577	0.979626486
0.96069909	0.585	0.993208829
0.96106898	0.596	1.01188455
**0.99574625**	0.597	**1.013582343**
1	0.6	1
0.90355376	0.611	1.018333333
0.97695998	0.595	0.991666667
0.97760667	0.595	0.991666667
**0.9678312**	0.616	**1.026666667**
1	0.648	1
1.05466453	0.63	0.972222222
0.97500668	0.651	1.00462963
1.00748463	0.648	1
**0.99719326**	0.647	**0.99845679**
1	0.648	1
0.98230474	0.647	0.99845679
0.9931462	0.646	0.99691358
0.98133898	0.65	1.00308642
**0.98485934**	0.651	**1.00462963**
1	0.647	1
0.91384615	0.654	1.010819165
0.92446154	0.653	1.00927357
1.02107692	0.658	1.017001546
**0.98261538**	0.656	**1.013910355**
1	0.661	1
0.96442061	0.664	1.004538578
0.98043511	0.664	1.004538578
0.97114368	0.665	1.006051437
**0.96940625**	0.664	**1.004538578**
1	0.66	1
0.99700939	0.662	1.003030303
0.98372199	0.661	1.001515152
1.00352059	0.657	0.995454545
**0.98099637**	0.662	**1.003030303**
1	0.653	1
0.95657196	0.652	0.998468606
0.96930997	0.652	0.998468606
0.96764347	0.653	1
**0.98473748**	0.654	**1.001531394**
1	0.48	1
1.22919842	0.464	0.966666667
1.1127705	0.484	1.008333333
1.08259677	0.47	0.979166667
**1.05067053**	0.473	**0.985416667**
